# Partial Least Squares Enhances Genomic Prediction of New Environments

**DOI:** 10.3389/fgene.2022.920689

**Published:** 2022-07-08

**Authors:** Osval A. Montesinos-López, Abelardo Montesinos-López, Kismiantini, Armando Roman-Gallardo, Keith Gardner, Morten Lillemo, Roberto Fritsche-Neto, José Crossa

**Affiliations:** ^1^ Facultad de Telemática, Universidad de Colima, Colima, Mexico; ^2^ Centro Universitario de Ciencias Exactas e Ingenierías (CUCEI), Universidad de Guadalajara, Guadalajara, Mexico; ^3^ Statistics Study Program, Universitas Negeri Yogyakarta, Yogyakarta, Indonesia; ^4^ International Maize and Wheat Improvement Center (CIMMYT), Texcoco, Mexico; ^5^ Department of Plant Sciences, Norwegian University of Life Sciences, IHA/CIGENE, Ås, Norway; ^6^ Laboratory of Allogamous Plant Breeding, Genetics Department, Luiz de Queiroz College of Agriculture, University of São Paulo, Piracicaba, Brazil; ^7^ Colegio de Postgraduados, Montecillo, Mexico

**Keywords:** Bayesian genomic-enabled prediction, genotype x environment interaction, partial least squares, disease data, Bayesian analysis, partial least squares, maize and wheat data

## Abstract

In plant breeding, the need to improve the prediction of future seasons or new locations and/or environments, also denoted as “leave one environment out,” is of paramount importance to increase the genetic gain in breeding programs and contribute to food and nutrition security worldwide. Genomic selection (GS) has the potential to increase the accuracy of future seasons or new locations because it is a predictive methodology. However, most statistical machine learning methods used for the task of predicting a new environment or season struggle to produce moderate or high prediction accuracies. For this reason, in this study we explore the use of the partial least squares (PLS) regression methodology for this specific task, and we benchmark its performance with the Bayesian Genomic Best Linear Unbiased Predictor (GBLUP) method. The benchmarking process was done with 14 real datasets. We found that in all datasets the PLS method outperformed the popular GBLUP method by margins between 0% (in the Indica data) and 228.28% (in the Disease data) across traits, environments, and types of predictors. Our results show great empirical evidence of the power of the PLS methodology for the prediction of future seasons or new environments.

## Introduction

Genomic selection (GS) proposed by Meuwissen et al. (2001) is a disruptive methodology that uses statistical machine learning algorithms and data to improve the selection of candidate lines early in time without the need to measure phenotypic information. The empirical evidence in favor of GS methodology can be found in applications of many crops, such as wheat, maize, cassava, rice, chickpea, groundnut, etc. (Roorkiwal et al., 2016; Crossa et al., 2017; Wolfe et al., 2017; Huang et al., 2019). However, the practical implementation of GS is challenging because the GS methodology does not always guarantee medium or high prediction accuracies, since many factors affect the prediction performance.

One important complexity of GS models arises when predicting unphenotype cultivars in specific environments (e.g., planting data-site-management combinations) by incorporating G×E interaction into the genomic-based statistical models. Also important is the genomic complexity related to G×E interactions for multi-traits, as these interactions require statistical-genetic models that exploit the complex multivariate relations due to multi-trait and multi-environment variance-covariance but also to exploit the genetic correlations between environments, between traits, and between traits and environments simultaneously. In GS modeling, the interaction between markers and environmental covariates can be a complex task due to the high dimensionality of the matrix of markers, environmental covariates, or both. Jarquín et al. (2014) suggested modeling this interaction using Gaussian processes, where the associated variance-covariance matrix induces a reaction norm model. The authors showed that assuming normality for the terms involving the interaction and also assuming that the interaction obtained using a first-order multiplicative model is distributed normally, then the covariance function is the cell-by-cell product (Hadamard) of two covariance structures, one describing the genetic information and the other describing the environmental effects. Many studies using a reaction norm model indicated that including the G×E interaction in the model substantially increased the accuracy of across-environment (locations and/or years) predictions (Crossa et al., 2017).

Many of the factors that affect the prediction performance of the GS methodology are under scrutiny to be improved. For example, the quality of the marker data is of paramount importance and the breeder needs to be careful to use only high-quality markers. Also, the quality of the training population is key to guaranteeing reasonable prediction accuracies in untested new lines. For this reason, research is in progress to optimize the design of the training-testing sets. Regarding statistical machine learning algorithms, there is also research in progress to select the best algorithm for each implementation. Currently, it is very common to find applications in GS using random forest, mixed models, Bayesian methods (GBLUP, BRR, BayesA, BayesB, BayesC, and Bayes Lasso), support vector machine, gradient boosting machine methods, and deep learning methods. But, as stated in the “No Free Lunch Theorem,” none of these algorithms is the best statistical machine learning algorithm for predictive modeling problems such as classification and regression. The no free lunch theorem indicates that the performance of all statistical machine learning algorithms is similar in select specific situations. However, there are niches where a particular algorithm consistently outperforms the others. For example, in interacting with images, there is empirical evidence that deep neural network methods are the best, yet one limitation of deep neural network models is that they require considerably large datasets (Montesinos-López O. A. et al., 2018; Montesinos-López A. et al., 2018; Montesinos-López et al., 2019).

The Bayesian method GBLUP is quite robust and most of the time produces reasonable and competitive predictions with the advantage that we can implement this method requiring no extra time for the tuning process. This is due to its efficient default hyperparameters for most applications, which is an important advantage regarding support vector machine, gradient boosting machine, random forest, and deep learning models that require considerable effort to select a reasonable set of hyperparameters. However, it is important to note that deep learning methods are the most demanding and challenging for selecting reasonable hyperparameters since this algorithm requires many inputs (Montesinos-López O. A. et al., 2018; Montesinos-López A. et al., 2018).

For the prediction of new environments (or seasons), most statistical machine learning methods struggle to produce reasonable predictions because often there is not a good match between the distribution of the training and testing set. For this reason, the prediction of a new season or environments is a more challenging task than when using conventional strategies of cross-validation (CV1), which tests the performance of lines that have not been measured in any of the observed environments, and CV2, which tests the performance of lines that have been measured in some environments but not in others (Burgueño et al., 2012). This is significant for small plant breeding programs, where data from multiple locations are scarce or non-existent, with the need to predict which lines are more likely to perform better in future seasons or environments (Monteverde et al., 2019).

In multi-environmental plant breeding field trials, information on environments may enhance the information in genotype×environment interactions (GE), and the principal component regression procedure that relates environments to the principal component scores of the GE has been proposed (Aastveit and Martens, 1986). To overcome some of the interpretation difficulties present in the principal component regression scores, Aastveit and Martens (1986) proposed the partial least squares (PLS) regression method as a more direct and parsimonious linear model. PLS regression describes GE in terms of differential sensitivity of cultivars to environmental variables where explanatory variables are linear combinations of the complete set of measured environmental and/or cultivar variables with no limit to the number of explanatory covariables. Vargas et al. (1998, 1999) carried out extensive studies for assessing the importance of environmental covariables to interpret and understand GE in multi-environment plant breeding trials. Furthermore, Crossa et al. (1999) demonstrated the usefulness of PLS for understanding GE using environmental and marker covariables.

Based on the above considerations, in this study we explore the power of PLS for the prediction of new environments, a problem denoted as “leave one environment out.” The prediction accuracy of the PLS regression method is compared to that of GBLUP, one of the most robust and widely used methods in genomic selection. The benchmarking was done with 14 real datasets in which at least two environments were evaluated.

## Materials and Methods

### Bayesian GBLUP Model

The model used was

$$Y_{ij}=\mu +L_{i}+g_{j}+gL_{ij}+\epsilon_{ij}$$ (1)

where $L_{i}$ are the random effects of locations (environments) distributed as $E={\left(E_{1},\ldots ,E_{I}\right)}^{T}∼N_{I}\left(0,\sigma_{E}^{2}H\right)$ , where H is the environmental relationship matrix as computed by VanRaden (2008), but in place of using genomic information, it was computed using environmental variables; that is, $H=\frac{X_{E}X_{E}^{T}}{r}$ , where $X_{E}$ is the standardized (centered and scaled) matrix of dimension I×r containing the environmental information of I environments and for each environment were measured r environmental covariates; $g_{j},$ j=1,…,J , are the random effects of lines, $gL_{ij}$ are the random effects of location-line interaction (GE) and $\epsilon_{ij}$ are random error components in the model assumed to be independent normal random variables with mean **0** and variance $\sigma^{2}$ . Furthermore, it is assumed that $g={\left(g_{1},\ldots ,g_{J}\right)}^{T}∼N_{J}\left(0,\sigma_{g}^{2}G\right)$ , $\mathrm{gL}={\left(gL_{11},\ldots ,gL_{1J},\ldots ,\;gL_{IJ}\right)}^{T}∼N_{IJ}\left(0,\sigma_{gL}^{2}\left(H⊗G\right)\right)$ **,** where G is the genomic relationship matrix as computed by VanRaden (2008), ⊗ denotes the Kronecker product and H is the environmental relationship matrix of size I . Environmental covariates were available for only the last two datasets (datasets 13 and 14), so for the first 12 datasets the H environmental relationship matrix was reduced to an identity matrix, $I_{I},\;$ of dimension I×I . The implementation of this model was done in the BGLR library of Pérez and de los Campos (2014). This model contains GE but was also implemented without GE interaction, that is, the model (1) without the fourth component on the right side of Eq. 1, such that

$$Y_{ij}=\mu +L_{i}+g_{j}+\epsilon_{ij}$$ (2)

### Partial Least Squares (PLS) Method

PLS regression was first introduced by Wold (1966) and was originally developed for econometrics and chemometrics. It is a multivariate statistical technique designed to deal with the p>n problem, i.e., when the number of explanatory variables ( p ) is much larger (and more highly correlated) than the number of observations ( n ). The PLS works for relating one or more response variables (Y) to a set of explanatory variables ( X ) (Wold, 2001; Boulesteix and Strimmer, 2006).

For PLS regression, the components, called Latent Variables (LVs) in this context, are obtained iteratively. One starts with the SVD of the cross-product matrix $S=X^{T}Y$ , thereby including information on the variation in both X and Y , and on the correlation between them. The first left and right singular vectors, w and q , are used as weight vectors for X and Y , respectively, to obtain scores t and u :

t=Xw=Ew (3)

u=Yq=Fq (4)

where E and F are initialized as both X and Y , respectively. The X scores t are often normalized:

$$t=t/\sqrt{t^{T}t}$$ (5)

The Y scores u are not actually necessary in the regression but are often saved for interpretation purposes. Next, X and Y loadings are obtained by regressing against the same vector t :

$$p=E^{T}t$$ (6)

$$q=F^{T}t$$ (7)

Finally, the data matrices are “deflated”: the information related to this latent variable, in the form of the outer products $tp^{T}$ and $tq^{T}$ , is subtracted from the (current) data matrices E and F .

$$E_{n+1}=E_{n}-\;tp^{T}$$ (8)

$$F_{n+1}=F_{n}-\;tq^{T}$$ (9)

The estimation of the next component can then start from the SVD of the cross-product matrix $E_{n+1}^{T}F_{n+1}$ . After every iteration, vectors w , t , p and q are saved as columns in matrices W , T , P , and Q , respectively. One complication is that columns of matrix W cannot be compared directly: they are derived from successively deflated matrices E and F . It has been shown that an alternative way to represent the weights is that all columns relate to the original X matrix is given by

$$R=W{\left(P^{T}W\right)}^{-1}$$ (10)

Now, instead of regressing Y on X , we use T scores to calculate the regression coefficients, and later convert these back to the realm of the original variables by pre-multiplying with matrix **R** (since T=XR ):

$$B=R\;b=RQ^{T}$$ (11)

with $b={\left(T^{T}T\right)}^{-1}T^{T}Y$ . Again, here only the first a components are used. Since regression and dimension reduction are performed simultaneously, all B , T , W , P , and Q are part of the output. Both X and Y are considered when calculating the LV in T . Moreover, they are defined so that the covariance between the LV and the response is maximized. Finally, predictions for new data $\left(X_{\mathrm{new}}\right)$ should be made as:

$${\hat{Y}}_{\mathrm{new}}=X_{\mathrm{new}}B=X_{\mathrm{new}}\mathrm{Rb}=T_{\mathrm{new}}b$$ (12)

with $T_{\mathrm{new}}=X_{\mathrm{new}}R$ . How many components are optimal must be determined, usually by cross-validation. We used the root mean squared error of prediction (RMSEP), which was minimized with 10-fold cross-validation in the training dataset and for each value of LV (Mevik and Cederkvist, 2004).

PLS is similar to principal component regression (PCR). In theory, PLS regression should have an advantage over PCR. One could imagine a situation where a minor component in X is highly correlated with Y ; not selecting enough components would then lead to poor predictions. In PLS regression, such a component would be automatically present in the first LV. However, there is hardly any difference between the use of PLS regression and PCR; in most situations, the methods achieve similar prediction accuracies, although PLS regression usually needs fewer latent variables than PCR. Put the other way around: with the same number of latent variables, PLS regression will cover more of the variation in Y and PCR will cover more of X . Both behave similarly to ridge regression.

It is important to point out that under the PLS regression method, first we computed the design matrices (dummy variables) of environments $\left(X_{L}\right)$ , genotypes $\left(X_{g}\right)$ , and GE interactions $\left(X_{gL}\right)$ . The dimensions of matrices $X_{L}$ , $X_{g}$ , and $X_{gL}$ were JI×I , JI×J , and JI×JI , respectively. While the dimension of the response variable ( Y ), was of JI×1. Then, to include the environmental and markers information in the design matrices of environments $\left(X_{L}\right)$ , genotypes $\left(X_{g}\right)$ , and GE $\left(X_{gL}\right)$ , we used the following augmented design matrices: $X_{L}L_{E},\;X_{g}L_{g}$ , and $X_{gL}\left(L_{E}⊗L_{g}\right)$ for environments, the genotypes and GE components, respectively. $L_{E}$ is a matrix of order I×I that denotes the square root of the environmental relationship matrix  H or order I×I , while $L_{g}$ is a matrix of order J×J that denotes the square root of the genomic relationship matrix G or order J×J . It is important to point out that the resulting augmented matrices**:** $X_{L}L_{E},\;X_{g}L_{g}$ **,** and $X_{gL}\left(L_{E}⊗L_{g}\right)$ have the same dimension of the non-augmented matrices $X_{L}$ , $X_{g}$ , and $X_{gL}$ , since the matrices for which they were post-multiplied were square matrices that have the same number of columns of $X_{L}$ , $X_{g}$ , and $X_{gL}$ , respectively. For this reason, the input matrix used in the PLS analysis was $X=\left[X_{L}L_{E},\;X_{g}L_{g},\;X_{gL}\left(L_{E}⊗L_{g}\right)\right]$ when the predictor contained the GE term, which is of order JI×(I+J+JI) , but when the GE term was not included, the input matrix used was $X=\left[X_{L}L_{E},\;X_{g}L_{g}\right]$ of order JI×(I+J) . Both models (GBLUP and PLS) were implanted under a univariate (uni-trait) framework, so the response variable is a vector that contains information of all environments available in the dataset. Implementation of both models (GBLUP and PLS) was done in the R statistical software **(** R Core Team, 2022). However, the PLS model was implemented using the PLS library (Mevik and Wehrens, 2007).

### Data Sets

Note that for datasets 1-12, the PLS included only marker covariables, thus the data augmentation $X_{g}L_{g}$ applies and the markers are included as the square root of the genomic relationship matrix G $\left(L_{g}\right)$ . For datasets 13-14, the PLS included both marker and environmental covariables; thus, data augmentation applies to both factors $X_{L}L_{E},\;\text{and}\;X_{g}L_{g}$ where the environments are included as the square root of the environmental relationship matrix H $\left(L_{E}\right)$ .

#### Datasets 1-3. Elite Wheat Yield Trial (EYT) Years 2013–2014 and 2014-2015, 2015-2016

Three datasets were collected by the Global Wheat Program (GWP) of the International Maize and Wheat Improvement Center (CIMMYT) and belong to elite yield trials (EYT) established in four different cropping seasons with four or five environments. Dataset 1 is from 2013-2014, dataset 2 is from 2014-2015, and dataset 3 is from 2015-2016. The EYT datasets 1, 2 and 3 contain 776, 775, and 964 lines, respectively. The experimental design used was an alpha-lattice design and the lines were sown in 39 trials, each covering 28 lines and 2 checks in 6 blocks with 3 replications. Several traits were available for some environments and lines in each dataset. In this study, we included four traits measured for each line in each environment: days to heading (DTHD, number of days from germination to 50% spike emergence), days to maturity (DTMT, number of days from germination to 50% physiological maturity or the loss of the green color in 50% of the spikes), plant height, and grain yield (GY). For full details of the experimental design and how the Best Linear Unbiased Estimates (BLUEs) were computed, see Juliana et al. (2018).

The lines examined in datasets 2 and 3 were evaluated in five environments, while dataset 1 was evaluated in four. For EYT dataset 1, the environments were bed planting with five irrigations (Bed5IR), early heat (EHT), flat planting, and five irrigations (Flat5IR), and late heat (LHT). For EYT dataset 2, the environments were bed planting with two irrigations (Bed2IR), Bed5IR, EHT, Flat5IR and LHT, while for dataset 3, the environments evaluated were Bed2IR, Bed5IR, Flat5IR, flat planting with drip irrigation (FlatDrip), and LHT.

Genome-wide markers for the 2,515 (776 + 775+964) lines in the two datasets were obtained using genotyping-by-sequencing (GBS) (Elshire et al., 2011; Poland et al., 2012) at Kansas State University using an Illumina HiSeq2500. After filtering, 2,038 markers were obtained from an initial set of 34,900 markers. The imputation of missing marker data was carried out using LinkImpute (Money et al., 2015) and implemented in TASSEL (Trait Analysis by Association Evolution and Linkage) version 5 (Bradbury et al., 2007). Lines that had more than 50% missing data were removed, and 2,515 lines were used in this study (776 lines in the first dataset, 775 lines in the second dataset, and 964 lines in the third dataset).

#### Dataset 4. Groundnut Data

The phenotypic dataset reported by Pandey et al. (2020) contains information on the phenotypic performance for various traits in four environments. In the present study, we assessed predictions using the trait seed yield per plant (SYPP), pods per plant (NPP), pod yield per plant (PYPP) and yield per hectare (YPH), for 318 lines in four environments, denoted as Environment1 (ENV1): Aliyarnagar_Rainy 2015; Environment2 (ENV2): Jalgoan_Rainy 2015; Environment3 (ENV3): ICRISAT_Rainy 2015; Environment4 (ENV4): ICRISAT Post-Rainy 2015.

The dataset is balanced, resulting in a total of 1,272 assessments with each line included once in each environment. Marker data were available for all lines and 8,268 SNP markers remained after quality control (each marker was coded with 0, 1 and 2).

#### Dataset 5. Maize Data

This maize dataset was included in Souza et al. (2017) from USP (Universidad Sao Paulo) and consists of 722 (with 722 ×4 = 2,888 observations) maize hybrids obtained by crossing 49 inbred lines. The hybrids were evaluated in four environments (Env1-Env4) in Piracicaba and Anhumas, São Paulo, Brazil, in 2016. The hybrids were evaluated using an augmented block design, with two commercial hybrids as checks to correct for micro-environmental variation. At each site, two levels of nitrogen (N) fertilization were used. The experiment conducted under ideal N conditions received 100 kg ha-1 of N (30 kg ha-1 at sowing and 70 kg ha-1 in a coverage application) at the V8 plant stage, while the experiment with low N received 30 kg/ha of N at sowing. The parent lines were genotyped with an Affymetrix Axiom Maize Genotyping Array of 616 K SNPs. Markers with a minor allele frequency (MAF) of 0.05 were removed. After applying QC, 54,113 SNPs were available to make the predictions.

#### Dataset 6. Disease Data

This dataset contains 438 lines for which three diseases were recorded: *Pyrenophora tritici-repentis* (PTR) that causes a disease originally named “yellow spot” but also known as tan spot, yellow leaf spot, yellow leaf blotch, or helminthosporiosis. The second disease, *Parastagonospora nodorum* (SN), is a major fungal pathogen of wheat fungal taxon that includes several plant pathogens affecting the leaves and other parts of the plants. The third disease, *Bipolaris sorokiniana* (SB), is the cause of seedling diseases, common root rot and spot blotch of several crops such as barley and wheat. The 438 wheat lines were evaluated in the greenhouse for several replicates, and the replicates were considered as different environments (Env1, Env2, Env3, Env4, Env5, and Env6). The total number of observations was 438 ×6 = 2,628 observations for which the three traits were measured.

DNA samples were extracted from each line, following the manufacturer’s protocol. DNA samples were genotyped using 67,436 single nucleotide polymorphisms (SNPs). For a given marker, the genotype for the *i*th line was coded as the number of copies of a designated marker-specific allele carried by the *i*th line (absence = zero and presence = one). SNP markers with unexpected heterozygous genotypes were recoded as either AA or BB. We kept those markers that had fewer than 15% missing values. Next, we imputed the markers using observed allelic frequencies. We also removed markers with MAF<0.05. After quality control and imputation, a total of 11,617 SNPs were available for analysis.

#### Datasets 7-12. Wheat Data

Spring wheat lines selected for grain yield analyses from CIMMYT first year yield trials (YT) were used as the training population to predict the quality of lines selected from EYT for grain yield analyses in a second year. The analyses were conducted for only the grain yield trait unless specified, and using six sets of data, as reported below:

- Wheat_1 (2013-14/2014-15), 1,301 lines from the 2013-14 YT and 472 lines from the 2014-2015 EYT trial. In this dataset, only the grain yield trait was used.
- Wheat_2 (2014-15/2015-16), 1,337 lines from the 2014-15 YT and 596 lines from the 2015-2016 EYT trial.
- Wheat_3 (2015-16/2016-17), 1,161 lines from the 2015-16 YT and 556 lines from the 2016-2017 EYT trial.
- Wheat_4 (2016-17/2017-18), 1,372 lines from the 2016-17 YT and 567 lines from the 2017-2018 EYT trial.
- Wheat_5 (2017-18/2018-19), 1,386 lines from the 2017-18 YT and 509 lines from the 2018-2019 EYT trial.
- Wheat_6 (2018-19/2019-20), 1,276 lines from the 2018-19 YT and 124 lines from the 2019-2020 EYT trial. More details of these datasets can be found in Ibba et al. (2020).

All the lines were genotyped using genotyping-by-sequencing (GBS; Poland et al., 2012). The TASSEL version 5 GBS pipeline was used to call marker polymorphisms (Glaubitz et al., 2014), and a minor allele frequency of 0.01 was assigned for SNP discovery. The resulting 6,075,743 unique tags were aligned to the wheat genome reference sequence (RefSeq v.1.0) (IWGSC 2018) with an alignment rate of 63.98%. After filtering for SNPs with homozygosity >80%, *p*-value for Fisher’s exact test <0.001 and χ^2^ value lower than the critical value of 9.2, we obtained 78,606 GBS markers that passed at least one of those filters. These markers were further filtered for less than 50% missing data, greater than a 0.05 minor allele frequency, and less than 5% heterozygosity. Markers with missing data were imputed using the “expectation-maximization” algorithm in the “R” package rrBLUP (Endelman, 2011).

#### Datasets 13. Indica

The rice dataset, Monteverde et al. (2019), contains information on the phenotypic performance of four traits (GY = Grain Yield, PHR = Percentage of Head Rice Recovery, GC = percentage of Chalky Grain, PH = Plant Height) in three environments (2010, 2011, and 2012). For each year, 327 lines were evaluated and measured three times (once for each developmental stage: vegetative, reproductive, and maturation) and for 18 environmental covariates. 1) ThermAmp denotes the thermal amplitude (°C), average of daily thermal amplitude calculated as max temperature (°C)—min temperature (°C). 2) RelSun denotes the relative sunshine duration (%), quotient between the real duration of the brightness of the Sun and the possible geographical or topographic duration. 3) SolRad denotes solar radiation (cal/cm2/day), solar radiation calculated using Armstrong’s formula. 4) EfPpit denotes effective precipitation (mm), average daily precipitation in mm added and stored in the soil. 5) DegDay denotes degrees day in rice (°C), mean of daily average temperature minus 10°. 6) RelH denotes relative humidity (hs), sum of hours (0–24 h) where the relative humidity was equal to 100%. 7) PpitDay denotes the precipitation day, which is the sum of days when it rained. 8) MeanTemp denotes the mean temperature (°C), average of temperature over 24 h (0–24 h). 9) AvTemp denotes average temperature (°C), average temperature calculated as daily (Max + Min)/2. 10) MaxTemp denotes the maximum temperature (°C), average of maximum daily temperature. 11) MinTemp denotes minimum temperature (°C), average of minimum daily temperature. 12) TankEv denotes the tank water evaporation (mm), amount of evaporated water under influence of Sun and wind. 13) Wind denotes wind speed (2 m/km/24 h), distance covered by wind (in km) over 2 m height in 1 day. 14) PicheEv denotes piche evaporation (mm), the amount of evaporated water without the influence of the Sun. 15) MinRelH denotes the minimum relative humidity (%), lowest value of relative humidity for the day. 16) AccumPpit denotes accumulated precipitation (mm), daily accumulated precipitation. 17) Sunhs denotes sunshine duration, sum of total hours of sunshine per day. 18) MinT15 denotes the minimum temperature below 15°, the sum of the days where the minimum temperature was below 15°.

This dataset is balanced, giving a total of 981 assessments with each line included once in each environment. Marker data were available for all lines and 16,383 SNP markers remained after quality control (each marker was coded with 0, 1, and 2).

#### Datasets 14. Japonica

This rice dataset was also reported by Monteverde et al. (2019) but belongs to the tropical Japonica population and contains phenotypic information on the same four traits as for the Indica population (GY = Grain Yield, PHR = Percentage of Head Rice Recovery, GC = percentage of Chalky Grain, PH = Plant Height) in five environments (2009, 2010, 2011, 2012, and 2013). In 2009, 2010, 2011, 2012, and 2013, 93, 292, 316, 316, and 134 lines were evaluated, respectively. In each year, the same 54 environmental covariates measured in the Indica dataset (dataset 13) were examined, that is, the 18 covariates measured in the three developmental stages: vegetative, reproductive, and maturation. Since this dataset is not balanced, a total of 1,051 assessments were evaluated in the five environments. Marker data were available for 320 lines and 16,383 SNP markers remained after quality control (each marker was coded with 0, 1, and 2).

### Metrics for Evaluation of Prediction Accuracy

We employed the leave-one-environment out type of cross-validation in each of the 14 datasets (Montesinos-López et al., 2022). That is, we used I−1 environments as the training set and the remaining environments as the testing set until each of the I environments played the role of testing one time. In the case of the model (*1*), we did not require a tuning process, but under PLS we divided the respective training (information of the I−1 environments) in inner training (80% of the training) and validation set (20% of the training). This was done under five nested cross-validations, which implies the training set was divided into five parts and one of those was used as the validation set and the remaining four as inner training. Then, the average of the five validation sets was reported as prediction accuracy to select the optimal hyperparameter, which in PLS, was the number of principal components to retain (Montesinos-López et al., 2022). Then, with this optimal hyperparameter (number of principal components), we refitted the model with the whole training set (information of the I−1 environments), and finally, with these trained models, the prediction for the testing set (a full environment) was obtained. As a metric to evaluate the prediction accuracy, we used the normalized root mean square error $\left(NRMSE=\frac{RMSE}{\bar{y}}\right)$ , where $RMSE=\sqrt{\frac{1}{T}(\overset{T}{\underset{i=1}{\sum }}{\left(y_{i}-\hat{f}(x_{i})\right)}^{2}}$ , with $y_{i}\;$ denoting the observed value i , while $\hat{f}\left(x_{i}\right)$ represents the predicted value for observation i , with i=1,…,n) . This metric was computed under the GBLUP ( $NRMSE_{GBLUP}$ ) and PLS $\left(NRMSE_{PLS}\right)$ methods, and then we calculated with both metrics the relative efficiency:

$$RE_{NRMSE}=\frac{NRMSE_{GBLUP}}{NRMSE_{PLS}}$$

When $RE_{NRMSE}>1,\;$ the best prediction performance in terms of NRMSE was obtained using the PLS method, but when $RE_{NRMSE}<1,$ the GBLUP method was superior in terms of prediction accuracy, and of course, when $RE_{NRMSE}=1,$ both methods were equally efficient.

## Results

The results are given in nine sections. In sections 1–6 are the results for dataset 1 to dataset 6, respectively, section 7 gives the results for the datasets (dataset 7 to dataset 12), section 8 gives the results for dataset 13, and section 9 gives the results for dataset 14.

### Dataset 1. EYT_1

#### With Predictor = E + G + GE

**FIGURE 1:**
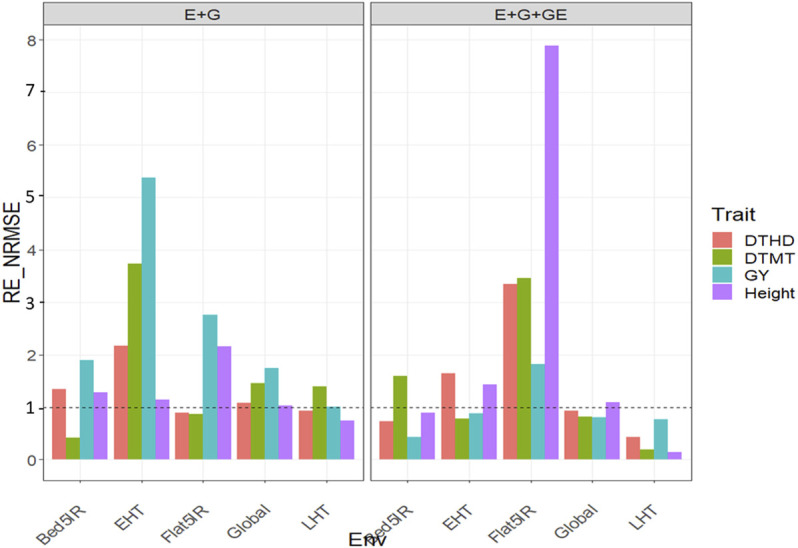
Relative efficiency in terms of normalized root mean square error (RE_NRMSE) computed by dividing the NRMSE under the best linear unbiased predictor model (GBLUP) between the NRMSE of the partial least squares regression method. Prediction performance is reported for each environment and across environments (Global) in dataset 1 (EYT_1), also with two predictors (E + G and E + G + GE). When the RE_NRMSE>1 the PLS outperforms in terms of prediction performance (lower NRMSE) the GBLUP method.

When the GE was considered in the predictor, we observed relative efficiencies in terms of NRMSE of the GBLUP methods vs. the PLS method for trait DTHD of 0.73, 1.642, 3.343, and 0.434 for environments Bed5IR, EHT, Flat5IR, and LHT, respectively. Only in environments EHT and Flat5IR genomic prediction performance of PLS regression method was superior to GBLUP by 64.2% (EHT) and 234.3% (Flat5IR). However, across environments, we observed that the GBLUP method was better than the PLS since the relative efficiency was equal to 0.929. Across environments, the GBLUP outperformed the PLS by 7.1% in terms of prediction performance (Figure 1 with predictor = E + G + GE). For trait DTMT, the $RE_{NRMSE}$ were 1.591 (Bed5IR), 0.779 (EHT), 3.457 (Flat5IR) and 0.197 (LHT). Only in environments Bed5IR and Flat5IR the PLS outperformed the GBLUP by 59.1% and 245.7%, respectively, while in the remaining two of four environments, the GBLUP method was the best. Across environments, the GBLUP method was also better than the PLS by 17.7% ( $RE_{NRMSE}$ = 0.823) (Figure 1 with predictor = E + G + GE). Also, in trait GY, only in one out of four environments the PLS outperformed the GBLUP method, since the $RE_{NRMSE}$ were 0.435 (Bed5IR), 0.884 (EHT), 1.825 (Flat5IR) and 0.764 (LHT). Across environments, the GBLUP was better than the PLS by 18.9% since the $RE_{NRMSE}$ = 0.811 (Figure 1 with predictor = E + G + GE). Finally, for trait Height, the relative efficiencies were 0.889 (Bed5IR), 1.431 (EHT), 7.895 (Flat5IR), 0.145 (LHT), and 1.095 (Global), which means that the PLS outperformed the GBLUP method by 43.1% (EHT), 689.5% (Flat5IR), and by 9.5% across environments. But in environments like Bed5IR and LHT, the GBLUP method was superior to the PLS method by 11.1% and 85.5%, respectively (Figure 1 with predictor = E + G + GE). See more details in Supplementary Material.

#### With Predictor = E + G

When only the main effects of environments and genotypes were considered in the predictor, the $RE_{NRMSE}$ for each environment and across environments for trait DTHD were 1.344 (Bed5IR), 2.175 (EHT), 0.892 (Flat5IR), 0.930 (LHT), and 1.087 (Global), so the PLS outperformed the GBLUP method by 34.4% (Bed5IR), 117.5% (EHT), and by 8.7% across environments (Figure 1 with predictor = E + G). For trait DTMT, the $RE_{NRMSE}$ were 0.419 (Bed5IR), 3.738 (EHT), 0.875 (Flat5IR), 1.393 (LHT) and 1.455 (Global), so the PLS was better than the GBLUP method by 273.8%, 39.3% and 45.5% in EHT, LHT, and across environments, respectively (Figure 1 with predictor = E + G), while in trait GY, the $RE_{NRMSE}$ were 1.894 (Bed5IR), 5.372 (EHT), 2.758 (Flat5IR), 1.002 (LHT) and 1.745 (Global). The PLS outperformed the GBLUP by 89.4%, 437.2%, 175.8%, 0.2%, and 74.5% in Bed5IR, EHT, Flat5IR, LHT, and Global, respectively (Figure 1 with predictor = E + G). Finally, for trait Height, the relative efficiencies were 1.285 (Bed5IR), 1.143 (EHT), 2.160 (Flat5IR), 0.745 (LHT), and 1.036 (Global), meaning the PLS method outperformed the GBLUP method in three out of four environments and across environments by 28.5% (Bed5IR), 14.3 (EHT), 116% (Flat5IR), and 3.6% (across environments) (Figure 1 with predictor = E + G). See more details in Supplementary Material.

### Dataset 2. EYT_2

#### With Predictor = E + G + GE

**FIGURE 2:**
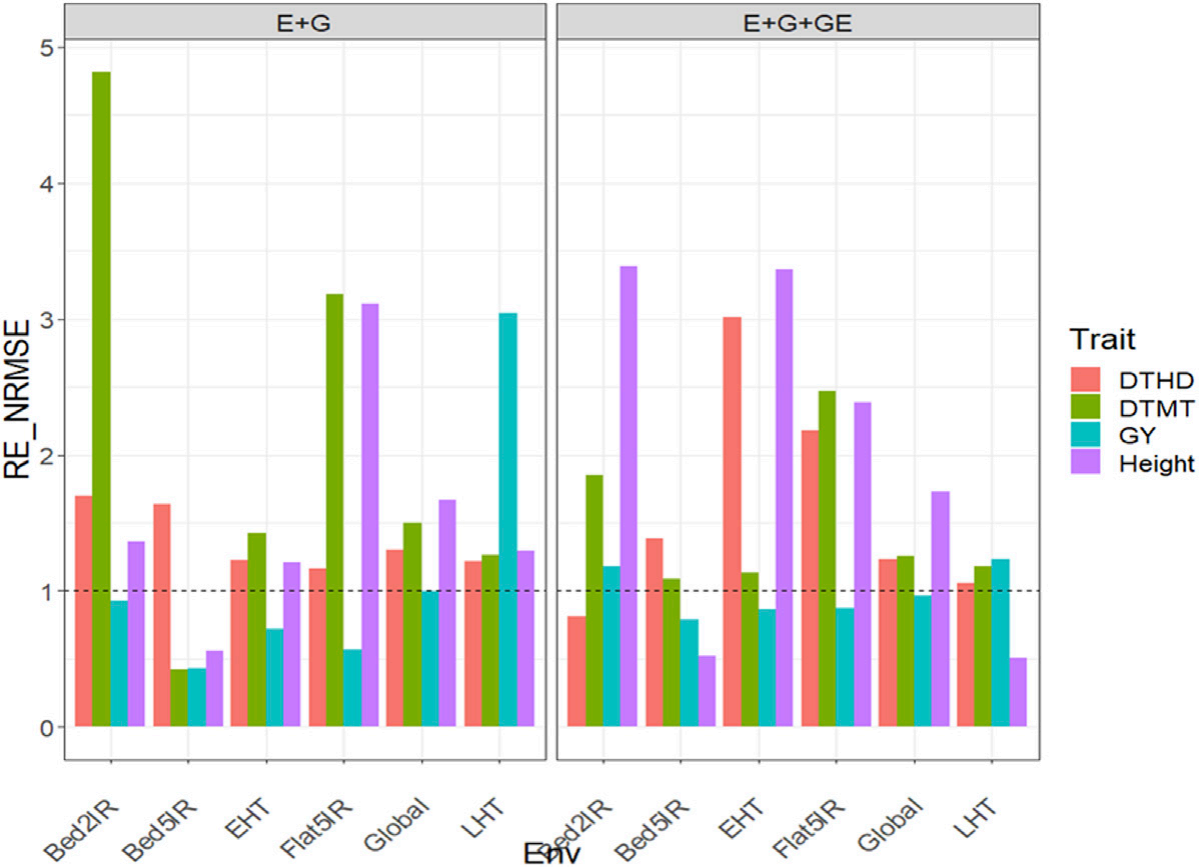
Relative efficiency in terms of normalized root mean square error (RE_NRMSE) computed by dividing the NRMSE under the best linear unbiased predictor model (GBLUP) between the NRMSE of the partial least squares regression method. Prediction performance is reported for each environment and across environments (Global) in dataset 2 (EYT_2), also with two predictors (E + G and E + G + GE). When the RE_NRMSE>1, the PLS outperforms the GBLUP method in terms of prediction performance (lower NRMSE).

With GE in the predictor, the $RE_{NRMSE}$ for trait **DTHD** were 0.81 (Bed2IR), 1.386 (Bed5IR), 3.011 (EHT), 2.181 (Flat5IR), 1.059 (LHT), and 1.235 (Global), which means that in four out of five environments, the PLS method was better than the GBLUP method by 38.6%, 201.1%, 118.1%, and 5.9% in environments Bed5IR, EHT, Flat5IR, and LHT, respectively. Across environments, the PLS outperformed the GBLUP method by 23.5% (Figure 2 with predictor = E + G + GE). For trait DTMT, the $RE_{NRMSE}$ were 1.85 (Bed2IR), 1.091 (Bed5IR), 1.135 (EHT), 2.467 (Flat5IR), 1.177 (LHT), and 1.255 (Global), which means that in the five environments, the PLS was superior to the GBLUB method by 85% (Bed2IR), 9.1% (Bed5IR), 13.5 (EHT), 146.7% (Flat5IR), 17.7 (LHT), and 25.5 (across environments) (Figure 2 with predictor = E + G + GE). In trait GY, the PLS method outperformed the GBLUP method only in two out of five environments by 17.8% (Bed2IR) and 23.2% (LHT), since the $RE_{NRMSE}$ were 1.178 (Bed2IR), 0.79 (Bed5IR), 0.866 (EHT), 0.875 (Flat5IR), 1.232 (LHT), and 0.966 (Global). For this reason, across environments, the GBLUP method was better than the PLS method by 3.4% (Figure 2 with predictor = E + G + GE). Finally, for trait Height, the relative efficiencies were 3.389 (Bed2IR), 0.526 (Bed5IR), 3.367 (EHT), 2.387 (Flat5IR), 0.509 (LHT) and 1.732 (Global), meaning that PLS was better than the GLUP method in three out of five environments, with gains of 238.9% (Bed2IR), 236.7% (EHT), 138.7% (Flat5IR), and 73.2% across environments (Figure 2 with predictor = E + G + GE). See more details in Supplementary Material.

#### With Predictor = E + G

Under this predictor for trait DTHD, the $RE_{NRMSE}$ efficiencies were 1.699 (Bed2IR), 1.636 (Bed5IR), 1.224 (EHT), 1.168 (Flat5IR), 1.214 (LHT), and 1.299 (Global). This means that in the five environments, the PLS outperformed the GBLUP method by 69.9% (Bed2IR), 63.6% (Bed5IR), 22.4% (EHT), 16.8% (Flat5IR), 21.4% (LHT), and 29.9% (Global) (Figure 2 with predictor = E + G). For trait DTMT, the PLS was better than the GBLUP method by 381.7% (Bed2IR), 42.6% (EHT), 217.8% (Flat5IR), 26.6% (LHT), and 49.7% (Global), since the $RE_{NRMSE}$ were 4.817 (Bed2IR), 0.424 (Bed5IR), 1.426 (EHT), 3.178 (Flat5IR), 1.266 (LHT), and 1.497 (Global) (Figure 2 with predictor = E + G), while in trait GY. The $RE_{NRMSE}$ were 0.931 (Bed2IR), 0.429 (Bed5IR), 0.724 (EHT), 0.571 (Flat5IR), 3.04 (LHT), and 0.998 (Global), so that the PLS outperformed the GBLUP only in environment LHT by 204% and also across environments, the GBLUP method was slightly better (0.2%) than the PLS (Figure 2 with predictor = E + G). Finally, for trait Height, the relative efficiencies were 1.359 (Bed2IR), 0.559 (Bed5IR), 1.209 (EHT), 3.114 (Flat5IR), 1.293 (LHT), and 1.665 (Global), which means that the PLS method outperformed the GBLUP method in four out of five environments and across environments by 35.9% (Bed2IR), 20.9% (EHT), 211.4% (Flat5IR), 29.3% (LHT), and 66.5% (Global) (Figure 2 with predictor = E + G). See more details in Supplementary Material.

### Dataset 3. EYT_3

#### With Predictor = E + G + GE

**FIGURE 3:**
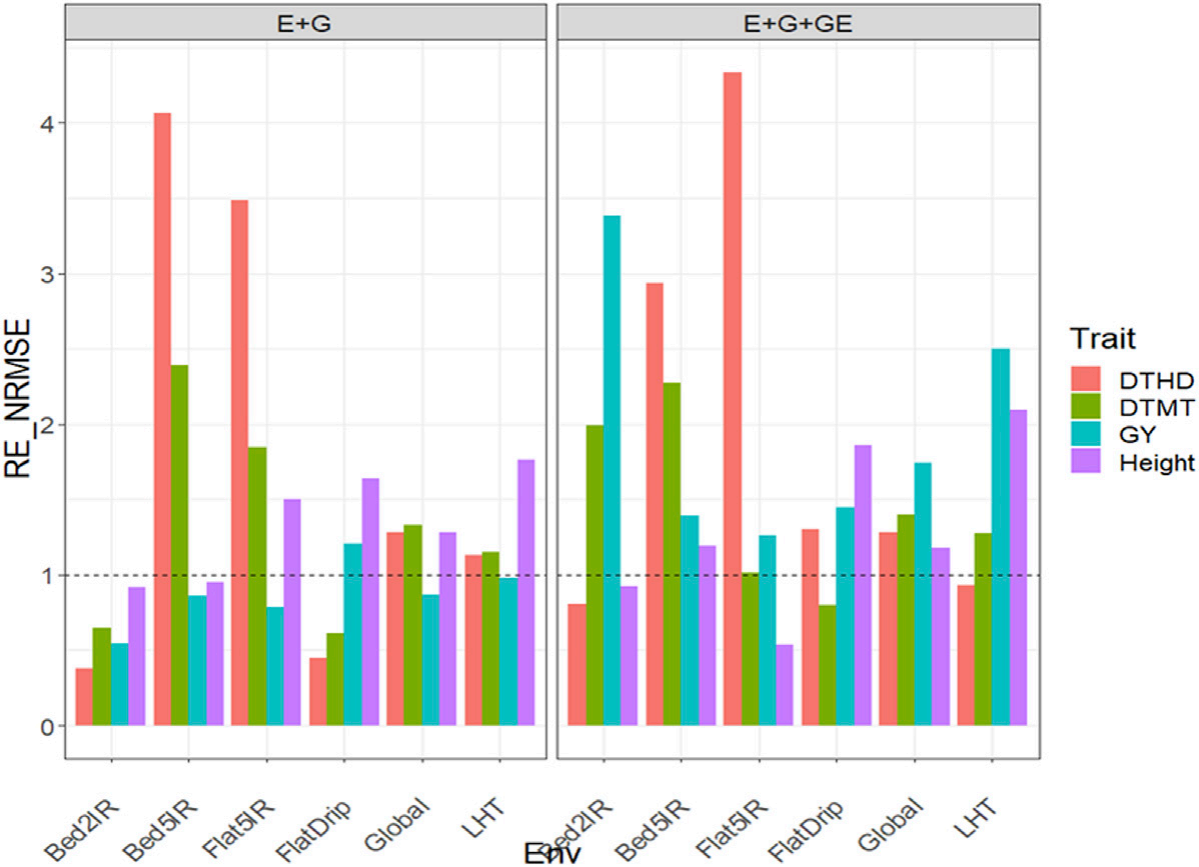
Relative efficiency in terms of normalized root mean square error (RE_NRMSE) computed by dividing the NRMSE under the best linear unbiased predictor model (GBLUP) between the NRMSE of the partial least squares regression method. Prediction performance is reported for each environment and across environments (Global) in dataset 3 (EYT_3), also with two predictors (E + G and E + G + GE). When the RE_NRMSE>1, the PLS outperforms the GBLUP method in terms of prediction performance (lower NRMSE).

Under this predictor, the relative efficiencies in terms of NRMSE for trait DTHD were 0.809, 2.935, 4.335, 1.299, 0.932, and 1.283 for Bed2IR, Bed5IR, Flat5IR, FlatDrip, LHT, and Global, respectively. The PLS was better than the GBLUP in three out of five environments and across environments by 193.5%, 233.5%, 29.9%, and 28.3% in Bed5IR, Flat5IR, FlatDrip, and Global, respectively (Figure 3 with predictor = E + G + GE). For trait DTMT, the $RE_{NRMSE}$ were 1.991(Bed2IR), 2.273 (Bed5IR), 1.01 (Flat5IR), 0.802 (FlatDrip), 1.274 (LHT), 1.396 (Global), so in four out of five environments, the PLS outperformed the GBLUP by 99.1% (Bed2IR), 127.3% (Bed5IR), 0.1% (Flat5IR), 27.4% (LHT), and 39.6% (Global) (Figure 3 with predictor = E + G + GE), while in trait GY, the $RE_{NRMSE}$ were 3.385 (Bed2IR), 1.393 (Bed5IR), 1.26 (Flat5IR), 1.444 (FlatDrip), 2.503 (LHT), and 1.741 (Global), which means that the PLS outperformed the GBLUP by 238.5% (Bed2IR), 39.3% (Bed5IR), 26% (Flat5IR), 44.4% (FlatDrip), 150.3% (LHT) and 74.1% (Global) (Figure 3 with predictor = E + G + GE). Finally, for trait Height, the relative efficiencies were 0.923 (Bed2IR), 1.192 (Bed5IR), 0.537 (Flat5IR), 1.858 (FlatDrip), 2.097 (LHT) and 1.181 (Global), which means that the PLS outperformed the GBLUP in only three environments and across environments by 19.2% (Bed5IR), 85.8% (FlatDrip), 109.7% (LHT) and 18.1% (Global) (Figure 3 with predictor = E + G + GE). See more details in Supplementary Material.

#### With Predictor = E + G

Under this predictor, the $RE_{NRMSE}$ for trait DTHD were 0.382, 4.064, 3.487, 0.448, 1.131, and 1.28 for Bed2IR, Bed5IR, Flat5IR, FlatDrip, LHT, and Global, respectively. The PLS was better than the GBLUP in three out of five environments and across environments by 306.4%, 248,7%, 13.1%, and 28% in Bed5IR, Flat5IR, LHT, and Global, respectively (Figure 3 with predictor = E + G). For trait DTMT, the $RE_{NRMSE}$ were 0.648 (Bed2IR), 2.388 (Bed5IR), 1.845 (Flat5IR), 0.614 (FlatDrip), 1.15 (LHT), and 1.329 (Global), so in three out of five environments, the PLS outperformed the GBLUP by 138.8% (Bed5IR), 84.5% (Flat5IR), 15% (LHT), and 32.9% (Global) (Figure 3 with predictor = E + G), while in trait GY, the $RE_{NRMSE}$ were 0.547 (Bed2IR), 0.863 (Bed5IR), 0.786 (Flat5IR), 1.205 (FlatDrip), 0.981 (LHT), and 0.865 (Global), which means that the PLS outperformed the GBLUP only in environment FlatDrip by 20.5%, while in the remaining four and across environments, the GBLUP outperformed the PLS method (Figure 3 with predictor = E + G). Finally, in trait Height, the PLS method outperformed the GBLUP method in three out of five environments since the relative efficiencies were 0.917 (Bed2IR), 0.953 (Bed5IR), 1.505 (Flat5IR), 1.641 (FlatDrip), 1.763 (LHT), and 1.281 (Global). That is, the gains of the PLS over the GBLUP method in the three environments and across environments were 50.5% (Flat5IR), 64.1% (FlatDrip), 76.3% (LHT), and 28.1% (Global) (Figure 3 with predictor = E + G). See more details in Supplementary Material.

### Dataset 4. Groundnut

#### With Predictor = E + G + GE

**FIGURE 4:**
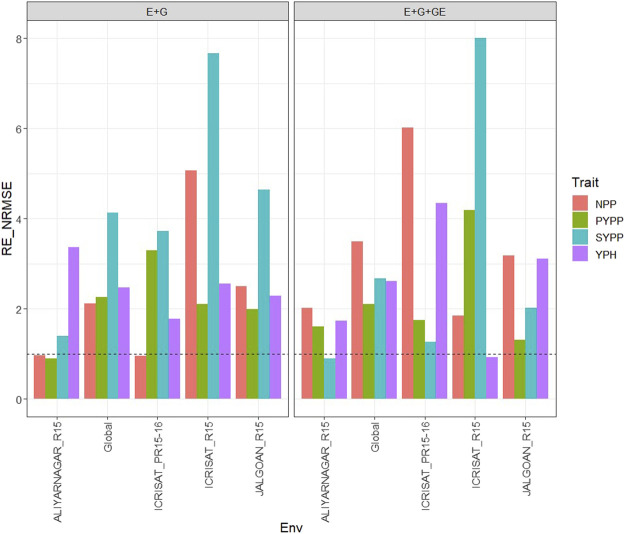
Relative efficiency in terms of normalized root mean square error (RE_NRMSE) computed by dividing the NRMSE under the best linear unbiased predictor model (GBLUP) between the NRMSE of the partial least squares regression method. Prediction performance is reported for each environment and across environments (Global) in dataset 4 (Groundnut), also with two predictors (E + G and E + G + GE). When the RE_NRMSE>1, the PLS outperforms the GBLUP method in terms of prediction performance (lower NRMSE).

With GE in the predictor, the observed $RE_{NRMSE}$ for trait NPP observed were 2.022 (ALIYARNAGAR_R15), 6.022 (ICRISAT_PR15-16), 1.853 (ICRISAT_R15), 3.177 (JALGOAN_R15), and 3.495 (Global), which means that in the four environments, the PLS method was better than the GBLUP method by 102.2%, 502.2%, 85.3%, 217.7%, and 249.5% in environments ALIYARNAGAR_R15, ICRISAT_PR15-16, ICRISAT_R15, JALGOAN_R15, and across environments, respectively (Figure 4 with predictor = E + G + GE). For trait PYPP, the $RE_{NRMSE}$ were 1.61 (ALIYARNAGAR_R15), 1.749 (ICRISAT_PR15-16), 4.183 (ICRISAT_R15), 1.304 (JALGOAN_R15) and 2.102 (Global), so that in the four environments, the PLS was superior to the GBLUB method by 61% (ALIYARNAGAR_R15), 74.9% (ICRISAT_PR15-16), 318.3 (ICRISAT_R15), 30.4 (JALGOAN_R15), and 110.2 (across environments) (Figure 4 with predictor = E + G + GE). In trait SYPP, the PLS method outperformed the GBLUP method in three out of four environments by 25.9% (ICRISAT_PR15-16), 700% (ICRISAT_R15), and 102.5% (JALGOAN_R15), since the relative efficiencies were 0.899 (ALIYARNAGAR_R15), 1.259 (ICRISAT_PR15-16), 8 (ICRISAT_R15), 2.025 (JALGOAN_R15), and 2.666 (across environments) (Figure 4 with predictor = E + G + GE). Finally, for trait YPH, the relative efficiencies were 1.74 (ALIYARNAGAR_R15), 4.345 (ICRISAT_PR15-16), 0.929 (ICRISAT_R15), 3.113 (JALGOAN_R15), and 2.617 (across environments). In three out of four environments and across environments, the best predictions were obtained with the PLS method, with gains over the GBLUP method of 74% (ALIYARNAGAR_R15), 334.5% (ICRISAT_PR15-16), 211.3% (JALGOAN_R15) and 161.7% (across environments) (Figure 4 with predictor = E + G + GE). See more details in Supplementary Material.

#### With Predictor = E + G

Without the GE term in the predictor, the observed $RE_{NRMSE}$ for trait NPP were 0.965 (ALIYARNAGAR_R15), 0.957 (ICRISAT_PR15-16), 5.072 (ICRISAT_R15), 2.494 (JALGOAN_R15), and 2.124 (Global), which means that in two out of four environments and across environments, the PLS method was better than the GBLUP method by 407.2% (ICRISAT_R15), 149.4% (JALGOAN_R15), and 112.4% (Global) (Figure 4 with predictor = E + G). For trait PYPP, the observed $RE_{NRMSE}$ were 0.894 (ALIYARNAGAR_R15), 3.291 (ICRISAT_PR15-16), 2.097 (ICRISAT_R15), 1.995 (JALGOAN_R15), and 2.263 (Global), which means that in three out of four environments and across environments, the PLS was superior to the GBLUB method by 229.1% (ICRISAT_PR15-16), 109.7% (ICRISAT_R15), 99.5% (JALGOAN_R15), and 126.3% (across environments) (Figure 4 with predictor = E + G). In trait SYPP, the PLS method outperformed the GBLUP method in the four environments by 40% (ALIYARNAGAR_R15), 271.6% (ICRISAT_PR15-16), 666.7% (ICRISAT_R15), 364.7% (JALGOAN_R15), and 313.9% (across environments), since the relative efficiencies were 1.4 (ALIYARNAGAR_R15), 3.716 (ICRISAT_PR15-16), 7.667 (ICRISAT_R15), 4.647 (JALGOAN_R15), and 4.139 (across environments) (Figure 4 with predictor = E + G). Finally, for trait YPH, the relative efficiencies were 3.364 (ALIYARNAGAR_R15), 1.777 (ICRISAT_PR15-16), 2.557 (ICRISAT_R15), 2.283 (JALGOAN_R15), and 2.475 (across environments). That is, in the four environments and across environments, the best predictions were obtained with the PLS method with gains over the GBLUP method of 236.4% (ALIYARNAGAR_R15), 77.7% (ICRISAT_PR15-16), 155.7% (ICRISAT_R15), 128.3% (JALGOAN_R15), and 147.5% (across environments) (Figure 4 with predictor = E + G). See more details in Supplementary Material.

### Dataset 5. Maize Data

#### With Predictor = E + G + GE

**FIGURE 5:**
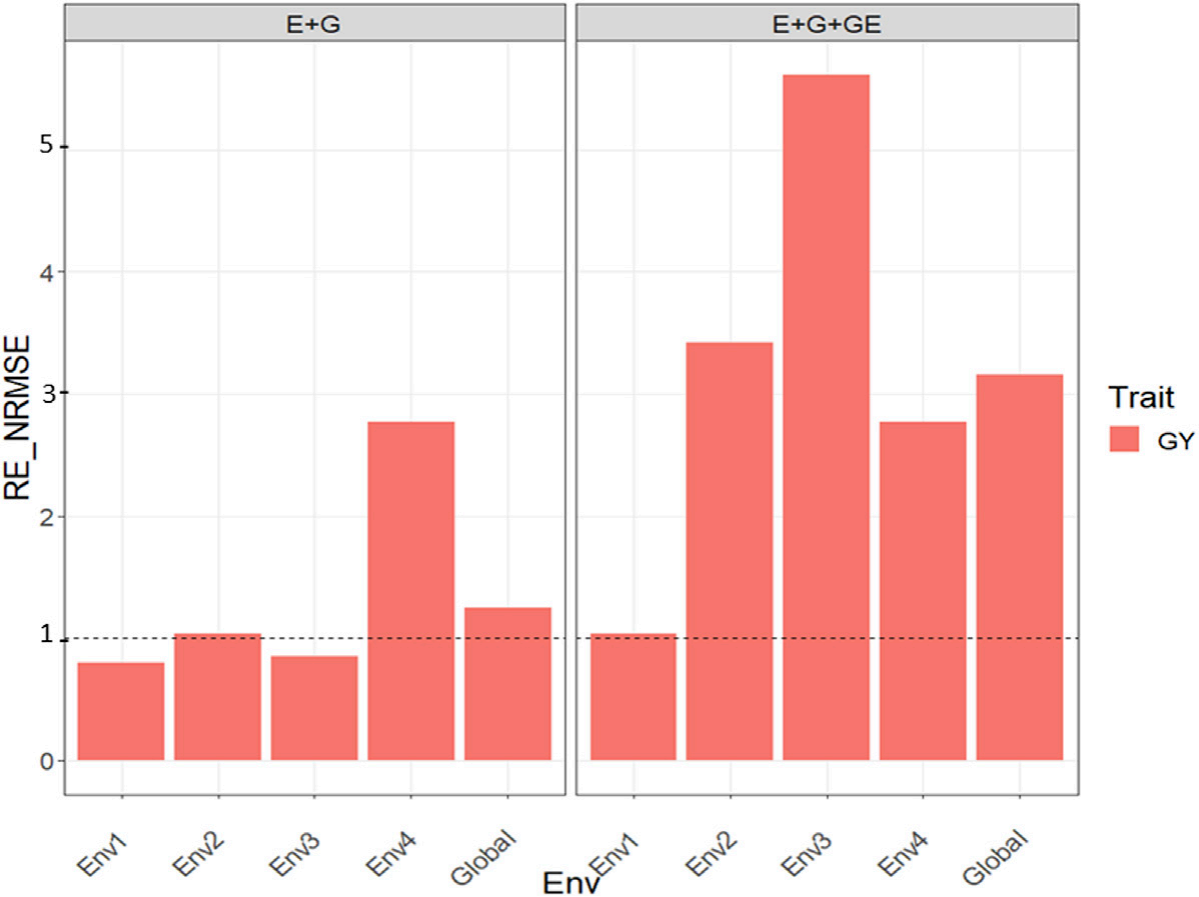
Relative efficiency in terms of normalized root mean square error (RE_NRMSE) computed by dividing the NRMSE under the best linear unbiased predictor model (GBLUP) between the NRMSE of the partial least squares regression method. Prediction performance is reported for each environment and across environments (Global) in dataset 5 (Maize), also with two predictors (E + G and E + G + GE). When the RE_NRMSE>1 the PLS outperforms the GBLUP method in terms of prediction performance (lower NRMSE).

With GE, the observed $RE_{NRMSE}$ for trait GY were 1.038 (Env1), 3.417 (Env2), 5.611 (Env3), 2.767 (Env4), and 3.155 (Global), which means in all environments the PLS method was better than the GBLUP method by 3.8% (Env1), 241.7% (Env2), 461.1% (Env3), 176.7% (Env4), and 215.5% across environments (Figure 5 with predictor = E + G + GE). See more details in Supplementary Material.

#### With Predictor = E + G

Without the GE, the observed $RE_{NRMSE}$ for trait GY were 0.801 (Env1), 1.041 (Env2), 0.854 (Env3), 2.772 (Env4), and 1.251 (Global), that is, only in two out of four environments and across environments, the PLS was superior to the GBLUP method, and the gains observed were 4.1% (Env2), 177.2% (Env4), and 25.1% across environments (Figure 5 with predictor = E + G). See more details in Supplementary Material.

### Dataset 6. Disease Data

#### With Predictor = E + G + GE

**FIGURE 6:**
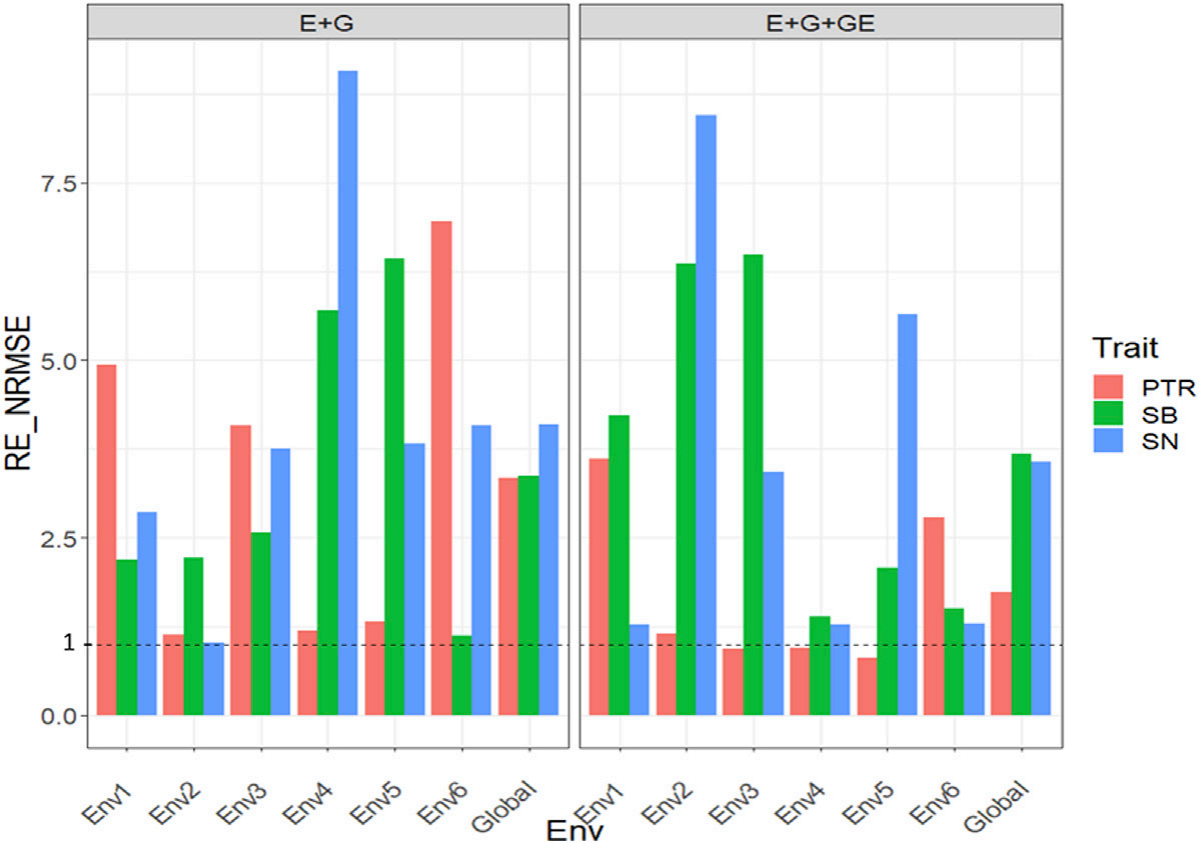
Relative efficiency in terms of normalized root mean square error (RE_NRMSE) computed by dividing the NRMSE under the best linear unbiased predictor model (GBLUP) between the NRMSE of the partial least squares regression method. Prediction performance is reported for each environment and across environments (Global) in dataset 6 (Disease), also with two predictors (E + G and E + G + GE). When the RE_NRMSE>1 the PLS outperforms the GBLUP method in terms of prediction performance (lower NRMSE).

With GE, the $RE_{NRMSE}$ for trait SN were 1.282 (Env1), 8.445 (Env2), 3.431 (Env3), 1.282 (Env4), 5.643 (Env5), 1.292 (Env6), and 3.57 (Global), which means that in the six environments and across environments, the PLS method was better than the GBLUP method by 28.2% (Env1), 744.5% (Env2), 243.1% (Env3), 28.2% (Env4), 464.3% (Env5), 29.2% (Env6), and 257% (Global) (Figure 6 with predictor = E + G + GE). In trait PTR, only in three out of the six environments, the PLS method outperformed the GBLUP method since the observed $RE_{NRMSE}$ were 3.615 (Env1), 1.157 (Env2), 0.932 (Env3), 0.951 (Env4), 0.807 (Env5), 2.788 (Env6), and 1.727 (Global). But, even though in only three out of six environments, PLS was better than the GBLUP method, across environments the PLS outperformed the GBLUP by 72.7% (Figure 6 with predictor = E + G + GE). However, in trait SB in all environments, the PLS was superior to the GBLUP method because the observed $RE_{NRMSE}$ were 4.221 (Env1), 6.36 (Env2), 6.491 (Env3), 1.388 (Env4), 2.078 (Env5), 1.506 (Env6), and 3.676 (Global). That is, the PLS outperformed the GBLUP method between 38% and 549.1% (Figure 6 with predictor = E + G + GE). See more details in Supplementary Material.

#### With Predictor = E + G

Without GE for trait SN, the PLS outperformed the GBLUP method between 0.17% and 807.2% since the observed $RE_{NRMSE}$ were 2.86 (Env1), 1.017 (Env2), 3.748 (Env3), 9.072 (Env4), 3.818 (Env5), 4.076 (Env6), and 4.09 (Global) (Figure 6 with predictor = E + G). In trait PTR, in the six environments, the PLS method outperformed the GBLUP method since the observed $RE_{NRMSE}$ were 4.936 (Env1), 1.14 (Env2), 4.08 (Env3), 1.194 (Env4), 1.315 (Env5), 6.952 (Env6), and 3.342 (Global) (Figure 6 with predictor = E + G), so that the PLS outperformed the GBLUP method across environments by 234.2% (Figure 6 with predictor = E + G + GE). However, in trait SB in all environments, the PLS was superior to the GBLUP method since the observed $RE_{NRMSE}$ were 2.19 (Env1), 2.214 (Env2), 2.566 (Env3), 5.703 (Env4), 6.434 (Env5), 1.124 (Env6), and 3.367 (Global). That is, the PLS outperformed the GBLUP method by 236.7% across environments (Figure 6 with predictor = E + G). See more details in Supplementary Material.

### Dataset 7-12. Wheat Data

#### With Predictor = E + G + GE

**FIGURE 7:**
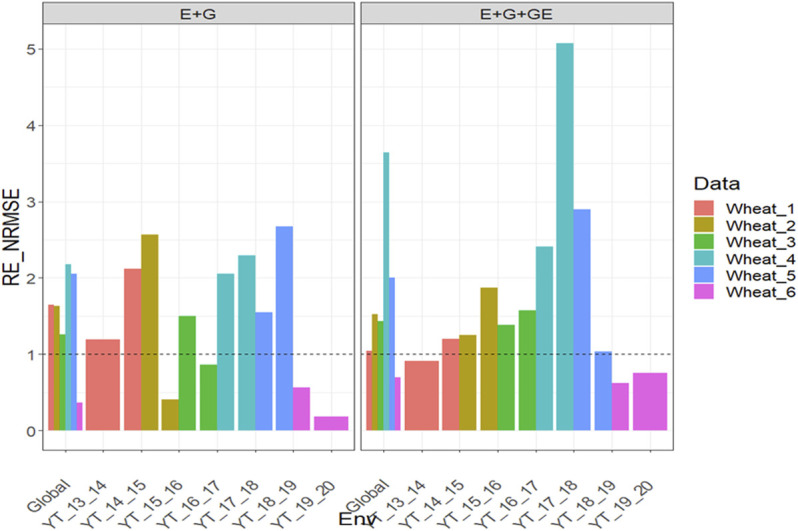
Relative efficiency in terms of normalized root mean square error (RE_NRMSE) computed by dividing the NRMSE under the best linear unbiased predictor model (GBLUP) between the NRMSE of the partial least squares regression method. Prediction performance is reported for each environment and across environments (Global) in datasets 7-12 (Wheat_1 to Wheat_6), also with two predictors (E + G and E + G + GE). When the RE_NRMSE>1, the PLS outperforms the GBLUP method in terms of prediction performance (lower NRMSE).

With GE, the $RE_{NRMSE}$ for dataset Wheat_1 were 0.915 (YT_13_14), 1.198 (YT_14_15), and 1.041 (Global), that is, the PLS outperformed the GBLUP method by 19.8% (YT_14_15), and 4.1% across environments (Figure 7 with predictor = E + G + GE). In Wheat_2, the $RE_{NRMSE}$ were 1.254 (YT_14_15), 1.875 (YT_15_16), and 1.526 (Global), that is, in the 2 years and across years, the best predictions were observed with the PLS method, and across years the gain of the PLS with regard to the GBLUP method was 52.6% (Figure 7 with predictor = E + G + GE). Also, in Wheat_3, the PLS outperformed the GBLUP method in the 2 years, and across years, the gain was 43.33%, since the $RE_{NRMSE}$ were 1.381 (YT_15_16), 1.573 (YT_16_17), and 1.433 (Global) (Figure 7 with predictor = E + G + GE). In Wheat_4 and Wheat_5, the PLS again outperformed the GBLUP method in the 2 years, and across years, the gain was 264.3% (Wheat_4) and 100.3% (Wheat_5**)** since the $RE_{NRMSE}$ in Wheat_4 were 2.41(YT_16_17), 5.073(YT_17_18) and 3.643 (Global), while in Wheat_5, they were 2.9 (YT_17_18), 1.033 (YT_18_19), 2.003 (Global) (Figure 7 with predictor = E + G + GE). Finally, in Wheat_6, the PLS did not outperform in either environment the GBLUP method since the $RE_{NRMSE}$ were 0.626 (YT_18_19), 0.754 (YT_19_20), and 0.694 (Global) (Figure 7 with predictor = E + G + GE). See more details in Supplementary Material.

#### With Predictor = E + G

With GE, the $RE_{NRMSE}$ for dataset Wheat_1 were 1.189 (YT_13_14), 2.122 (YT_14_15), and 1.648 (Global), that is, the PLS outperformed the GBLUP method by 18.9% (YT_14_15), 112.2% (YT_14_15), and 64.8% across environments (Figure 7 with predictor = E + G). In Wheat_2, the $RE_{NRMSE}$ were 2.563 (YT_14_15), 0.411 (YT_15_16) and 1.628 (Global), so that in only one year and across years the best predictions were observed by the PLS method, and across years the gain of the PLS with regard to the GBLUP method was 62.8% (Figure 7 with predictor = E + G). In Wheat_3, the PLS also outperformed the GBLUP method in 1 year, and across years the gain was 26.0%, since the $RE_{NRMSE}$ were 1.495 (YT_15_16), 0.865 (YT_16_17), and 1.26 (Global) (Figure 7 with predictor = E + G). In Wheat_4 and Wheat_5, the PLS outperformed the GBLUP method in the 2 years, and across years the gain was 117.5% (Wheat_4**)** and 105.1% (Wheat_5**)** since the $RE_{NRMSE}$ in Wheat_4 were 2.055 (YT_16_17), 2.296 (YT_17_18) and 2.175 (Global), while in Wheat_5 they were 1.55 (YT_17_18), 2.677 (YT_18_19), 2.051 (Global) (Figure 7 with predictor = E + G). Finally, in Wheat_6, in neither of the two environments the PLS outperformed the GBLUP method since the $RE_{NRMSE}$ were 0.567 (YT_18_19), 0.184 (YT_19_20) and 0.365 (Global) (Figure 7 with predictor = E + G). See more details in Supplementary Material.

### Dataset 13. Indica

#### With Predictor = E + G + GE

**FIGURE 8:**
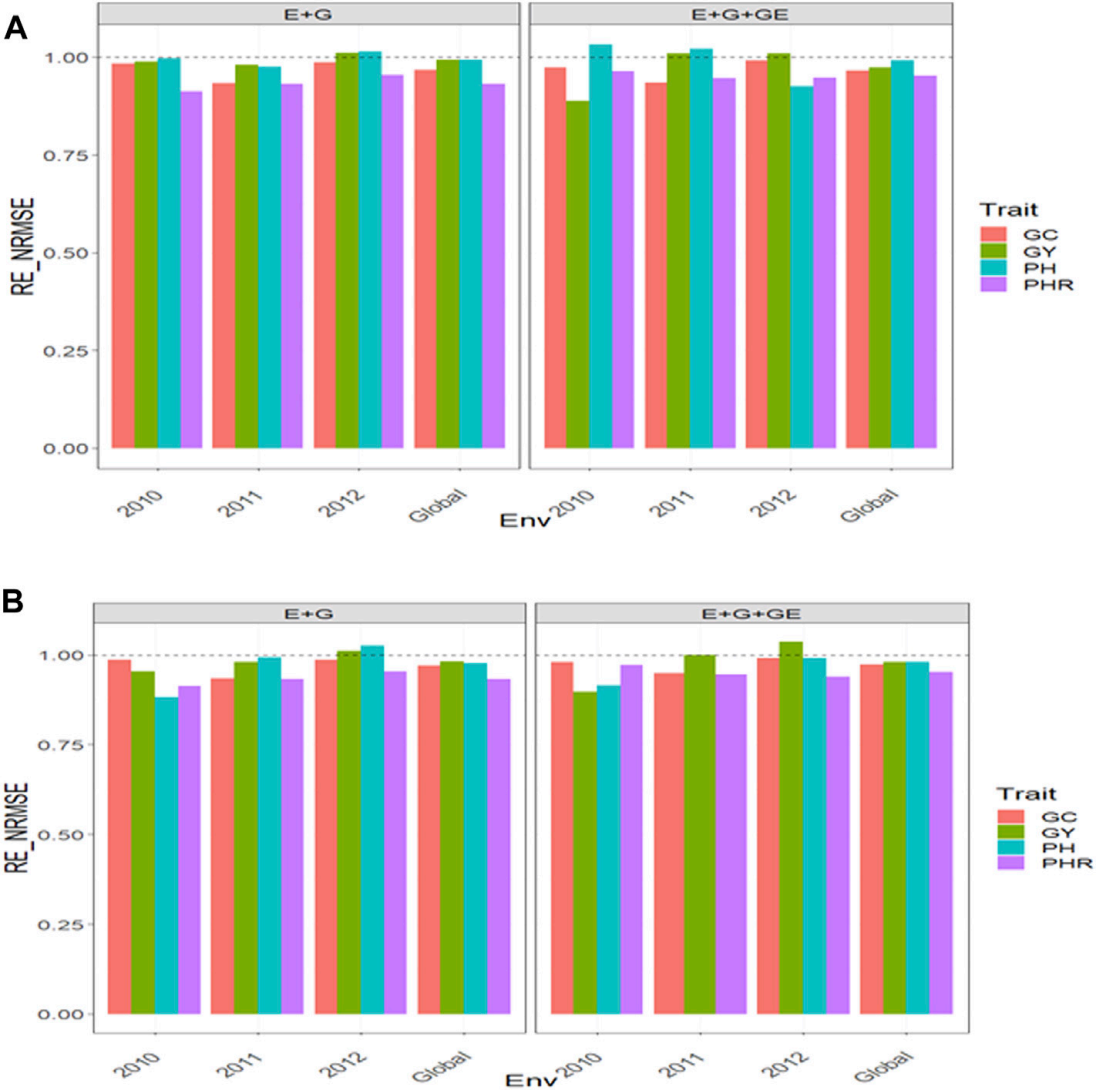
Relative efficiency in terms of normalized root mean square error (RE_NRMSE) computed by dividing the NRMSE under the best linear unbiased predictor model (GBLUP) between the NRMSE of the partial least squares regression method. Prediction performance is reported for each environment and across environments (Global) in dataset 13 (Indica), also with two predictors (E + G and E + G + GE). When the RE_NRMSE>1, the PLS outperforms the GBLUP method in terms of prediction performance (lower NRMSE). **(A)** With environmental covariates (EC) and **(B)** without EC.

With GE and considering the environmental covariates (EC), the $RE_{NRMSE}$ for trait GY were 0.888 in 2010, 1.010 in 2011, 1.010 in 2012, and 0.974 (Global), which means that in only one environment and across environments, the GBLUP method was better than the PLS method by 11.2% (2010) and 2.6% (Global). However, for this same trait but not including the environmental covariates, the $RE_{NRMSE}$ were 0.896 in 2010, 1.000 in 2011, 1.037 in 2012, and 0.980 (Global), so that the GBLUP only outperformed the PLS in 2010 by 10.4% and across years by 2% (Figure 8 with predictor = E + G + GE). For trait PHR, the environmental covariates in the predictor the $RE_{NRMSE}$ were 0.965 in 2010, 0.947 in 2011, 0.948 in 2012, and 0.953 (Global), which means that the GBLUP was better than the PLS by 3.5% in 2010, 5.3% in 2011, 5.2% in 2012, and 4.7% (Global). But ignoring the EC, the $RE_{NRMSE}$ were 0.972 in 2010, 0.947 in 2011, 0.940 in 2012, and 0.953 (Global), which means that the GBLUP was better than the PLS by 2.8% in 2010, 5.3% in 2011, 6.0% in 2012, and 4.7% (Global) (Figure 8 with predictor = E + G + GE).

For trait GC with EC, the $RE_{NRMSE}$ were 0.974 in 2010, 0.934 in 2011, 0.992 in 2012, and 0.966 (Global), which means that the GBLUP was better than the PLS by 2.6% in 2010, 6.63% in 2011, 0.8% in 2012, and 3.4% (Global). But ignoring the EC, the $RE_{NRMSE}$ were 0.979 in 2010, 0.949 in 2011, 0.992 in 2012, and 0.973 (Global), which means that the GBLUP was better than the PLS by 2.1% in 2010, 5.1% in 2011, 0.8% in 2012, and 2.7% (Global) (Figure 8 with predictor = E + G + GE). Finally, for trait PH with EC, the $RE_{NRMSE}$ were 1.031 in 2010, 1.02 in 2011, 0.925 in 2012, and 0.991 (Global), which means that the GBLUP was better than the PLS by 7.5% in 2012 and 0.9% (Global). But ignoring the EC, the $RE_{NRMSE}$ were 0.915 in 2010, 0.991 in 2012, and 0.979 (Global), which means that the GBLUP was better than the PLS by 8.5% in 2010, 0.9% in 2010, and 2.1% (Global) (Figure 8 with predictor = E + G + GE). See more details in Supplementary Material.

#### With Predictor = E + G

Without the GE and taking into account the EC, we observed the $RE_{NRMSE}$ for trait GY were 0.988 in 2010, 0.981 in 2011, 1.011 in 2012, and 0.993 (Global), which means that the GBLUP was better than the PLS by 1.2% in 2010, 1.9% in 2011, and 0.7% (Global). However, without taking into account the EC, the $RE_{NRMSE}$ were 0.955 in 2010, 0.980 in 2011, 1.011 in 2012, and 0.982 (Global), which means that the GBLUP was better than the PLS by 4.5% in 2010, 2.0% in 2011, and 1.8% (Global) (Figure 8 with predictor = E + G). For trait PHR, taking into account the EC, the $RE_{NRMSE}$ were 0.911 in 2010, 0.932 in 2011, 0.955 in 2012, and 0.932 (Global), that is, the GBLUP was better than the PLS by 8.9% in 2010, 6.8% in 2011, 4.5% in 2012, and 6.8% (Global). While ignoring the EC, the $RE_{NRMSE}$ were 0.913 in 2010, 0.933 in 2011, 0.954 in 2012, and 0.933 (Global), so that the GBLUP was better than the PLS by 8.7% in 2010, 6.7% in 2011, 4.6% in 2010, and 6.7% (Global) (Figure 8 with predictor = E + G).

For trait GC, taking into account the EC, the $RE_{NRMSE}$ were 0.983 in 2010, 0.933 in 2011, 0.986 in 2012, and 0.968 (Global), so that the GBLUP was better than the PLS by 1.7% in 2010, 6.7% in 2011, 1.4% in 2012, and 3.2% (Global). While ignoring the EC, the $RE_{NRMSE}$ were 0.987 in 2010, 0.935 in 2011, 0.986 in 2012, and 0.970 (Global), so that the GBLUP was better than the PLS by 1.3% in 2010, 6.5% in 2011, 1.4% in 2012, and 3.0% (Global). (Figure 8 with predictor = E + G). For trait PH, taking into account the EC, the $RE_{NRMSE}$ were 0.995 in 2010, 0.976 in 2011, 1.013 in 2012, and 0.992 (Global), so that the GBLUP was better than the PLS by 0.5% in 2010, 2.4% in 2011, and 0.8% (Global). While ignoring the EC, the $RE_{NRMSE}$ were 0.882 in 2010, 0.993 in 2011, 1.026 in 2012, and 0.977 (Global), so that the GBLUP was better than the PLS by 11.8% in 2010, 0.7% in 2011, and 2.3% (Global) (Figure 8 with predictor = E + G). See more details in Supplementary Material.

### Dataset 14. Japonica

#### With Predictor = E + G + GE

**FIGURE 9:**
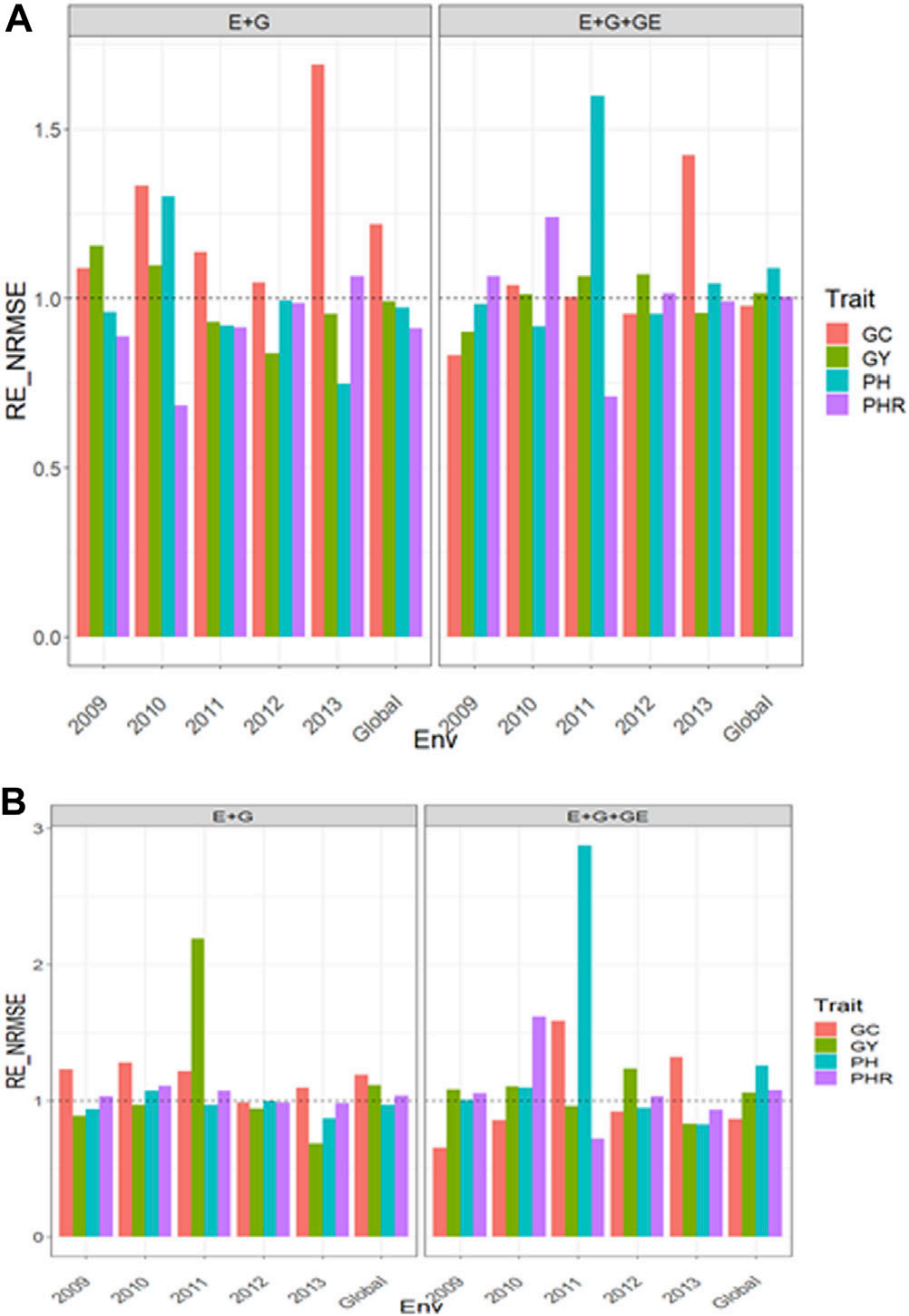
Relative efficiency in terms of normalized root mean square error (RE_NRMSE) computed by dividing the NRMSE under the best linear unbiased predictor model (GBLUP) between the NRMSE of the partial least squares regression method. Prediction performance is reported for each environment and across environments (Global) in dataset 14 (Japonica), also with two predictors (E + G and E + G + GE). When the RE_NRMSE>1, the PLS outperforms the GBLUP method in terms of prediction performance (lower NRMSE). **(A)** With environmental covariates (EC) and **(B)** without EC.

With GE the $RE_{NRMSE}$ for trait GY with (and without) EC were 0.900 (1.079), 1.012 (1.105), 1.064 (0.959), 1.071 (1.232), 0.957 (0.827), and 1.015 (1.059) for 2009, 2010, 2011, 2012, 2013, and Global, respectively. Which means that in both cases with (and without) EC across years, the best prediction performance was under the PLS method, which was superior by 1.5% with EC and by 5.9% without EC (Figure 9 with predictor = E + G + GE). While for trait PHR the $RE_{NRMSE}$ with (and without) EC were 1.065 (1.054), 1.240 (1.614), 0.709 (0.720), 1.015 (1.030), 0.991 (0.931), and 1.005 (1.077) for 2009, 2010, 2011, 2012, 2013, and Global, respectively. Which means that in both cases with (and without) EC across years, the PLS method outperformed the GBLUP method by 0.5% with EC and by 7.7% without EC (Figure 9 with predictor = E + G + GE). In trait GC the $RE_{NRMSE}$ with (and without) EC were 0.831 (0.654), 1.039 (0.853), 1.004 (1.585), 0.952 (0.917), 1.422 (1.321) and 0.977 (0.862) for 2009, 2010, 2011, 2012, 2013 and Global respectively, which means that with (and without) EC the GBLUP outperformed across year the PLS by 1.3% and 13.8% respectively (Figure 9 with predictor = E + G + GE). Finally, in trait PH the $RE_{NRMSE}$ with (and without) EC were 0.983 (1.004), 0.915 (1.095), 1.598 (2.872), 0.953 (0.944), 1.044 (0.824), 1.089 (1.257) for 2009, 2010, 2011, 2012, 2013, and Global, respectively, which means that with (and without) EC the PLS method outperformed across years the GBLUP method by 8.9 % and 25.7% respectively (Figure 9 with predictor = E + G + GE). See more details in Supplementary Material.

#### With Predictor = E + G

Without GE the $RE_{NRMSE}$ for trait GY, with (and without) EC were 1.156 (0.886), 1.096 (0.969), 0.929 (2.188), 0.838 (0.940), 0.954 (0.682), and 0.990 (1.111) for 2009, 2010, 2011, 2012, 2013 and Global, respectively, which means that without EC the PLS method outperformed across years the GBLUP method by 11.1%, but with EC the GBLUP outperformed the PLS by 1% (Figure 9 with predictor = E + G). In trait PHR the $RE_{NRMSE}$ with (and without) EC were 0.888 (1.031), 0.684 (1.108), 0.914 (1.072), 0.985 (0.984), 1.064 (0.979), and 0.911 (1.034) for 2009, 2010, 2011, 2012, 2013, and Global, respectively, which means that without EC the PLS method outperformed across years the GBLUP method by 3.4%, but with EC the GBLUP outperformed the PLS by 8.9% (Figure 9 with predictor = E + G). For trait GC the $RE_{NRMSE}$ with (and without) EC were 1.089 (1.228), 1.332 (1.279), 1.136 (1.215), 1.047 (0.986), 1.690 (1.094), and 1.218 (1.187) for 2009, 2010, 2011, 2012, 2013, and Global respectively, which means that with (and without) EC the PLS method outperformed across years the GBLUP method by 21.8 and 18.7%, respectively (Figure 9 with predictor = E + G). Finally, for trait PH, the $RE_{NRMSE}$ with (and without) EC were 0.959 (0.937), 1.302(1.070), 0.919 (0.969), 0.993(0.996), 0.746 (0.867), and 0.972 (0.968) for 2009, 2010, 2011, 2012, 2013, and Global, respectively, which means that with (and without) EC the GBLUP method outperformed across years the PLS method by 2.8 and 3.2%, respectively (Figure 9 with predictor = E + G). See more details in Supplementary Material.

## Discussion

Breeders need novel methodologies to match the productivity required to feed the world’s increasing population. For this reason, the adoption of genomic selection has been successful in plant breeding since it is a disruptive methodology that offers significant savings in resources in selecting candidate lines because GS is a predictive methodology trained with data that contain phenotypic and genotypic information. GS predicts breeding values or phenotypic values of new (untested) lines that were only genotyped, so it allows for earlier selection of lines, since we can obtain predictions for the lines of interest once the model is trained. (Crossa et al., 2017).

However, even though GS is efficient and simple to understand, breeders worldwide are still struggling to put it into practice successfully. To guarantee the successful implementation of GS, we need to guarantee moderate or high prediction accuracies, which is challenging since the resulting prediction accuracy depends on many factors, such as the degree of relatedness between the training and testing sets, the statistical machine learning model used, the size of the training and testing sets, the trait under study, the quality of inputs like markers and environmental information, and the prediction problem at hand (for example, the prediction of lines that were evaluated in other environments, or the prediction of lines that were not evaluated in previous years or environments, etc.).

The reaction norm model of Jarquín et al. (2014) has been successfully used for multi-environmental data and for incorporating interactions among multi-type input sources (e.g., dense molecular markers, pedigree, high-throughput phenotypes, environmental covariables) in several crops (Perez-Rodriguez et al., 2015; Crossa et al., 2016). One of the major gaps in GS research for G×E modeling using environmental data relies in the steps of collection, processing, and integrating those data into an ecophysiology-smart and parsimony manner. A method for envirotyping-informed genomic prediction of GxE using linear and non-linear kernels was presented by Costa-Neto et al. (2020) in two maize germplasms and taking account for additive, dominance and their interaction with environments. These authors showed that nonlinear kernels are the better option to deal with environmental similarity realized with environmental covariables in GS.

For the predictions mentioned above, research is underway to optimize the GS methodology so that its practical implementation can be more consistent, robust, and straightforward. For this reason, with the goal of finding more robust statistical machine learning methods, we evaluated the prediction performance of the partial least squares regression in the context of predicting a new year or environment (leave one environment out cross-validation strategy). We found the PLS method was superior to the popular GBLUP method in most cases when the goal was to predict a complete location or year.

Our results are encouraging because they show that PLS regression is a powerful tool for this type of prediction problem, since across all datasets, the PLS outperformed the GBLUP method by large margins (see Figures 1–7). Across traits, environments, and types of predictors, the PLS outperformed the GBLUP in terms of normalized root mean square error by 58.8% in dataset 1 (EYT_1 data), 52.52% in dataset 2 (EYT_2 data), 49.84% in dataset 3 (EYT_3 data), 178.21 in dataset 4 (groundnut data), 127.07% in dataset 5 (maize data), 228.28% in dataset 6 (disease data), 62.31% in datasets 7-12 (wheat_1 to wheat_6 datasets) and 0% in dataset 13 (Indica dataset). For those environments that were more similar to the environments in the training set less error of prediction was observed, while for those environments that were more different from the environments in the training set, more error of prediction was reached. Our findings agree with those reported by Monteverde et al. (2019) for this type of prediction problem (“leave one environment out”), but in our case, instead of using Pearson’s correlation as the metric of prediction performance, we used the normalized root mean square error.

Although our evaluations are empirical, we found that the PLS regression method is an efficient statistical machine learning prediction tool that is especially appropriate for small sample data with many (possibly correlated) independent variables, especially useful for small p and large n problems. However, since we only evaluated the leave one environment out cross-validation scheme, our assertions are valid only for this context. However, other authors (Campbell and Ntobedzi, 2007; Bergström et al., 2012; Vucicevic et al., 2015; Zhang et al., 2015; Kouskoura et al., 2019) have evaluated the prediction accuracy of the PLS methodology with conventional strategies of cross-validation (k-fold cross-validations, stratified-k-fold cross-validations, etc.) and there is also empirical evidence that the PLS regression method produces very competitive predictions.

We observed that implementing the PLS regression is fast for small datasets, but the larger the dataset, the more computational resources are required for its implementation. In general, we observed that the implementation of the PLS is at least two times slower than the GBLUP method. In part this is because under PLS selecting the optimal number of principal components (hyperparameters) is by means of an inner five-fold cross-validation that also is time consuming, but a key factor to a successful implementation of the PLS regression method. For this reason, the computational resources required for PLS implementation become a problem for moderate and large datasets. However, since the PLS regression only depends on one hyperparameter (number of principal components to retain), the tuning process is very easy, but with an increased need on computational resources.

One reason PLS is very competitive in terms of predictive modeling is that it automatically performs variable selection by creating the latent variables (factors) that are linear combinations of the original independent variables and the response variable (s). For this reason, PLS is quite efficient for handling many and correlated predictors, and additionally, it can detect which inputs (independent variables) are the most explanatory in a trained model by looking at the model coefficients (Wold, 2001; Mehmood et al., 2012). Vargas et al. (1998), Vargas et al. (1999) and Crossa et al. (1999) have shown the benefits of PLS for identifying the set of independent variables (environmental as well as molecular markers) that best explain the genotype by environment interaction. For all these reasons, PLS regression has become a well-established tool in predictive and association modeling in bioinformatics and genomics, because it is often possible to interpret the extracted factors in terms of the underlying physical system; to derive “hard” modeling information from the soft model.

It is important to note that the PLS regression method is not restricted only to continuous and univariate response variables since it can also be used for binary and categorical univariate response variables and to other tasks such as survival analysis, multivariate modeling, and modeling of regulation network. At present, most reported applications of the PLS method to genomic data focus on the analysis of microarray data from gene expression experiments.

## Conclusion

In this study, we compare the prediction performance of the popular GBLUP (in its Bayesian version) to the partial least squares (PLS) regression for the prediction of future seasons or new environments, when the goal is to predict the whole information of a new year or environment. This prediction scheme, denoted as “leave one environment out,” is challenging because many times we do not have any information or reference for the new year or environment we want to predict. Consequently, the predictions accuracies are low or very low. We found that the PLS regression outperformed the prediction performance of the Bayesian GBLUP method in terms of normalized root mean square error by at least 49.84% (across traits, environments, and types of predictors, when environmental covariates were not considered), which is an impressive large gain. For this reason, we encourage doing more research in this direction to support our findings. However, with our results, we can see that the PLS regression algorithm is a powerful tool for predicting new environments (or years) in the context of genomic selection.

## Data Availability

The original contributions presented in the study are included in the article/Supplementary Material; further inquiries can be directed to the corresponding authors.

## References

[B1] Aastveit A. H. Martens H. (1986). ANOVA Interactions Interpreted by Partial Least Squares Regression. Biometrics 42, 829–844. 10.2307/2530697

[B2] Bergström C. A. S. Charman S. A. Nicolazzo J. A. (2012). Computational Prediction of CNS Drug Exposure Based on a Novel *In Vivo* Dataset. Pharm. Res. 29, 3131–3142. 22744815 10.1007/s11095-012-0806-5

[B3] Boulesteix A.-L. Strimmer K. (2006). Partial Least Squares: a Versatile Tool for the Analysis of High-Dimensional Genomic Data. Briefings Bioinforma. 8, 32–44. 10.1093/bib/bbl016 16772269

[B4] Bradbury P. J. Zhang Z. Kroon D. E. Casstevens T. M. Ramdoss Y. Buckler E. S. (2007). TASSEL: Software for Association Mapping of Complex Traits in Diverse Samples. Bioinformatics 23, 2633–2635. 10.1093/bioinformatics/btm308 17586829

[B5] Burgueño J. de los Campos G. Weigel K. Crossa J. (2012). Genomic Prediction of Breeding Values when Modeling Genotype × Environment Interaction Using Pedigree and Dense Molecular Markers. Crop Sci. 52, 707–719. 10.2135/cropsci2011.06.0299

[B6] Campbell A. Ntobedzi A. (2007). Emotional Intelligence Coping and Psychological Distress: A Partial Least Square Approach to Developing a Predictive Model. Electron. J. Appl. Psychol. 3, 39–54. 10.7790/ejap.v3i2.91

[B7] Costa-Neto G. Fritsche-Neto R. Crossa J. (2020). Nonlinear Kernels, Dominance, and Envirotyping Data Increase the Accuracy of Genome-Based Prediction in Multi-Environment Trials. Heredity 126, 92–106. 10.1038/s41437-020-00353-1 32855544 PMC7852533

[B8] Crossa J. de los Campos G. Maccaferri M. Tuberosa R. Burgueño J. Pérez-Rodríguez P. (2016). Extending the Marker × Environment Interaction Model for Genomic-Enabled Prediction and Genome-wide Association Analysis in Durum Wheat. Crop Sci. 56, 2193–2209. 10.2135/cropsci2015.04.0260

[B9] Crossa J. Pérez-Rodríguez P. Cuevas J. Montesinos-López O. Jarquín D. de Los Campos G. (2017). Genomic Selection in Plant Breeding: Methods, Models, and Perspectives. Trends Plant Sci. 22 (11), 961–975. 10.1016/j.tplants.2017.08.011 28965742

[B10] Crossa J. Vargas M. van Eeuwijk F. A. Jiang C. Edmeades G. O. Hoisington D. (1999). Interpreting Genotype × Environment Interaction in Tropical Maize Using Linked Molecular Markers and Environmental Covariables. Theor. Appl. Genet. 99, 611–625. 10.1007/s001220051276 22665197

[B11] Elshire R. J. Glaubitz J. C. Sun Q. Poland J. A. Kawamoto K. Buckler E. S. (2011). A Robust, Simple Genotyping-By-Sequencing (GBS) Approach for High Diversity Species. PLoS One 6, e19379. 10.1371/journal.pone.0019379 21573248 PMC3087801

[B12] Endelman J. B. (2011). Ridge Regression and Other Kernels for Genomic Selection with R Package rrBLUP. Plant Genome 4, 250–255. 10.3835/plantgenome2011.08.0024

[B13] Glaubitz J. C. Casstevens T. M. Lu F. Harriman J. Elshire R. J. Sun Q. (2014). TASSEL-GBS: A High Capacity Genotyping by Sequencing Analysis Pipeline. PLoS ONE 9 (2), e90346. 10.1371/journal.pone.0090346 24587335 PMC3938676

[B14] Huang M. Balimponya E. G. Mgonja E. M. McHale L. K. Luzi-Kihupi A. Wang G.-L. (2019). Use of Genomic Selection in Breeding Rice (Oryza Sativa L.) for Resistance to Rice Blast (Magnaporthe Oryzae). Mol. Breed. 39, 114. 10.1007/s11032-019-1023-2

[B15] Ibba M. I. Crossa J. Montesinos-López O. A. Montesinos-López A. Juliana P. Guzman C. (2020). Genome-based Prediction of Multiple Wheat Quality Traits in Multiple Years. Plant Genome 13, e20034. 10.1002/tpg2.20034 33217204 PMC12806979

[B43] Jarquín D. Crossa J. Lacaze X. Du Cheyron P. Daucourt J. Lorgeou J. (2014). A Reaction Norm Model for Genomic Selection Using High-Dimensional Genomic and Environmental Data. Theor. Appl. Genet. 123 (7), 595–607. 10.1007/s00122-013-2243-1 PMC3931944 24337101

[B16] Juliana P., J. Singh R., P. Poland J. Mondal S. Crossa J. Montesinos-López O. A. (2018). Prospects and Challenges of Applied Genomic Selection-A New Paradigm in Breeding for Grain Yield in Bread Wheat. Plant Genome 11. (accepted). 10.3835/plantgenome2018.03.0017 PMC7822054 30512048

[B17] Kouskoura M. G. Piteni A. I. Markopoulou C. K. (2019). A New Descriptor via Bio-Mimetic Chromatography and Modeling for the Blood Brain Barrier (Part II). J. Pharm. Biomed. Analysis 164, 808–817. 10.1016/j.jpba.2018.05.021 29884296

[B18] Mehmood T. Liland K. H. Snipen L. Sæbø S. (2012). A Review of Variable Selection Methods in Partial Least Squares Regression. Chemom. Intelligent Laboratory Syst. 118, 62–69. 10.1016/j.chemolab.2012.07.010

[B19] Meuwissen T. H. E. Hayes B. J. Goddard M. E. (2001). Prediction of Total Genetic Value Using Genome-wide Dense Marker Maps. Genetics 157, 1819–1829. 10.1093/genetics/157.4.1819 11290733 PMC1461589

[B20] Mevik B.-H. Wehrens R. (2007). The Pls Package: Principal Component and Partial Least Squares Regression in R. J. Stat. Softw. 18 (2), 1–24. 10.18637/jss.v018.i02

[B21] Mevik B. r.-H. Cederkvist H. R. (2004). Mean Squared Error of Prediction (MSEP) Estimates for Principal Component Regression (PCR) and Partial Least Squares Regression (PLSR). J. Chemom. 18, 422–429. 10.1002/cem.887

[B22] Money D. Gardner K. Migicovsky Z. Schwaninger H. Zhong G.-Y. Myles S. (2015). LinkImpute: Fast and Accurate Genotype Imputation for Nonmodel Organisms. G3 Genes|Genomes|Genetics 5, 2383–2390. 10.1534/g3.115.021667 26377960 PMC4632058

[B23] Montesinos-López A. Montesinos-López O. A. Gianola D. Crossa J. Hernández-Suárez C. M. (2018b). Multi-environment Genomic Prediction of Plant Traits Using Deep Learners with a Dense Architecture. G3 Genes, Genomes, Genet. 8 (12), 3813–3828. 10.1534/g3.118.200740 PMC6288841 30291107

[B24] Montesinos-López O. A. Montesinos-López A. Crossa J. (2022). “Overfitting, Model Tuning and Evaluation of Prediction Performance,” in Multivariate Statistical Machine Learning Methods for Genomic Prediction. Editors Montesinos López O. A. Montesinos López A. Crossa J. (Cham, Switzerland: Springer International Publishing), 109–139.

[B25] Montesinos-López O. A. Montesinos-López A. Gianola D. Crossa J. Hernández-Suárez C. M. (2018a). Multi-trait, Multi-Environment Deep Learning Modeling for Genomic-Enabled Prediction of Plant. G3 Genes, Genomes, Genet. 8 (12), 3829–3840. 10.1534/g3.118.200728 PMC6288830 30291108

[B26] Montesinos-López O. A. Montesinos-López A. Tuberosa R. Maccaferri M. Sciara G. Ammar K. (2019). Multi-Trait, Multi-Environment Genomic Prediction of Durum Wheat with Genomic Best Linear Unbiased Predictor and Deep Learning Methods. Front. Plant Sci. 11 (10), 1–12. 10.3389/fpls.2019.01311 PMC6856087 31787990

[B27] Monteverde E. Gutierrez L. Blanco P. Pérez de Vida F. Rosas J. E. Bonnecarrère V. (2019). Integrating Molecular Markers and Environmental Covariates to Interpret Genotype by Environment Interaction in Rice (*Oryza Sativa* L.) Grown in Subtropical Areas. G3 (Bethesda) 9 (5), 1519–1531. PMID: 30877079; PMCID: PMC6505146. 10.1534/g3.119.400064 30877079 PMC6505146

[B28] Pandey M. K. Chaudhari S. Jarquin D. Janila P. Crossa J. Patil S. C. (2020). Genome-based Trait Prediction in Multi- Environment Breeding Trials in Groundnut. Theor. Appl. Genet. 133, 3101–3117. 10.1007/s00122-020-03658-1 32809035 PMC7547976

[B29] Pérez P. de los Campos G. (2014). BGLR: a Statistical Package for Whole Genome Regression and Prediction. Genetics 198 (2), 483–495. 25009151 10.1534/genetics.114.164442 PMC4196607

[B30] Pérez-Rodríguez P. Crossa J. Bondalapati K. De Meyer G. Pita F. Campos G. d. l. (2015). A Pedigree-Based Reaction Norm Model for Prediction of Cotton Yield in Multienvironment Trials. Crop Sci. 55, 1143–1151. 10.2135/cropsci2014.08.0577

[B31] Poland J. A. Brown P. J. Sorrells M. E. Jannink J.-L. (2012). Development of High-Density Genetic Maps for Barley and Wheat Using a Novel Two-Enzyme Genotyping-By-Sequencing Approach. PLoS One 7, e32253. 10.1371/journal.pone.0032253 22389690 PMC3289635

[B32] R Core Team (2022). R: A Language and Environment for Statistical Computing. Vienna. Austria: R Foundation for Statistical Computing.

[B33] Roorkiwal M. Rathore A. Das R. R. Singh M. K. Jain A. Srinivasan S. (2016). Genome-enabled Prediction Models for Yield Related Traits in Chickpea. Front. Plant Sci. 7, 1666. 10.3389/fpls.2016.01666 27920780 PMC5118446

[B34] Souza M. B. E. Cuevas J. Couto E. G. de. O. Pérez-Rodríguez P. Jarquín D. Fritsche-Neto R. (2017). Genomic-Enabled Prediction in Maize Using Kernel Models with Genotype × Environment Interaction. G3 (Bethesda) g3 7 (6), 1995–2014. 10.1534/g3.117.042341 28455415 PMC5473775

[B35] VanRaden P. M. (2008). Efficient Methods to Compute Genomic Predictions. J. dairy Sci. 91 (11), 4414–4423. 10.3168/jds.2007-0980 18946147

[B36] Vargas M. Crossa J. Eeuwijk F. A. Ramírez M. E. Sayre K. (1999). Using Partial Least Squares Regression, Factorial Regression, and AMMI Models for Interpreting Genotype × Environment Interaction. Crop Sci. 39 (4), 955–967. 10.2135/cropsci1999.0011183X003900040002x

[B37] Vargas M. Crossa J. Sayre K. Reynolds M. Ramírez M. E. Talbot M. (1998). Interpreting Genotype ✕ Environment Interaction in Wheat by Partial Least Squares Regression. Crop Sci. 38, 679–689. 10.2135/cropsci1998.0011183X003800030010x

[B38] Vucicevic J. Nikolic K. Dobričić V. Agbaba D. (2015). Prediction of Blood-Brain Barrier Permeation of α-adrenergic and Imidazoline Receptor Ligands Using PAMPA Technique and Quantitative-Structure Permeability Relationship Analysis. Eur. J. Pharm. Sci. 68, 94–105. 10.1016/j.ejps.2014.12.014 25542610

[B39] Wold H. (1966). “Estimation of Principal Components and Related Models by Iterative Least Sqares,” in Multivariate Analysis. Editor Krishnaiah P. R. (New York: Academic Press), 114–142.

[B40] Wold S. (2001). Personal Memories of the Early PLS Development. Chemom. Intelligent Laboratory Syst. 58 (2), 83–84. 10.1016/s0169-7439(01)00152-6

[B41] Wolfe M. D. Del Carpio D. P. Alabi O. Ezenwaka L. C. Ikeogu U. N. Kayondo I. S. (2017). Prospects for Genomic Selection in Cassava Breeding. Plant Genome 10, 15. 10.3835/plantgenome2017.03.0015 PMC7822052 29293806

[B42] Zhang Y.-H. Xia Z.-N. Yan L. Liu S.-S. (2015). Prediction of Placental Barrier Permeability: A Model Based on Partial Least Squares Variable Selection Procedure. Molecules 20, 8270–8286. 10.3390/molecules20058270 25961165 PMC6272791

