# Supplementary material for: Partial Least Squares Enhances Genomic Prediction of New Environments
Source: Front Genet. 2022 Jul 8;13:920689. doi: 10.3389/fgene.2022.920689 (PMC9608852; doi:10.3389/fgene.2022.920689)
Supplement: Supplementary file 1 [file DataSheet1.docx]

**APPENDICES**

**Appendix A**. Prediction performance for each environment and across environments (Global) of **dataset 1** (YET_1) in terms of mean square error (MSE), normalized root mean square error (NRMSE) and relative efficiency (RE) under two predictors (E+G; environments plus genotypes and E+G+GE, that also contain the genotype by environment interaction). MSE_PLS and MSE_G denote the MSE under the PLS and GBLUP method. NRMSE_PLS and NRMSE_G denote the NRMSE under the PLS and GBLUP method. RE_MSE and RE_NRMSE denote the RE computed with the MSE and NRMSE, respectively. RE were computed by dividing the prediction performance (with MSE or NRMSE) of the GBLUP method by the prediction performance of the PLS method.

| Data | Predictor | Trait | Env | MSE_PLS | NRMSE_PLS | MSE_G | NRMSE_G | RE_MSE | RE_NRMSE |
| --- | --- | --- | --- | --- | --- | --- | --- | --- | --- |
| EYT_1 | E+G+GE | DTHD | Bed5IR | 64.433 | 1.21 | 34.75 | 0.889 | 0.539 | 0.734 |
| EYT_1 | E+G+GE | DTHD | EHT | 53.751 | 0.906 | 144.859 | 1.488 | 2.695 | 1.642 |
| EYT_1 | E+G+GE | DTHD | Flat5IR | 10.041 | 0.949 | 112.219 | 3.174 | 11.177 | 3.343 |
| EYT_1 | E+G+GE | DTHD | LHT | 390.329 | 5.454 | 73.381 | 2.365 | 0.188 | 0.434 |
| EYT_1 | E+G+GE | DTHD | Global | 129.638 | 2.13 | 91.302 | 1.979 | 0.704 | 0.929 |
| EYT_1 | E+G+GE | DTMT | Bed5IR | 111.333 | 1.87 | 281.911 | 2.975 | 2.532 | 1.591 |
| EYT_1 | E+G+GE | DTMT | EHT | 126.905 | 1.523 | 77.012 | 1.186 | 0.607 | 0.779 |
| EYT_1 | E+G+GE | DTMT | Flat5IR | 18.143 | 1.42 | 216.831 | 4.908 | 11.952 | 3.457 |
| EYT_1 | E+G+GE | DTMT | LHT | 1248.22 | 8.158 | 48.398 | 1.606 | 0.039 | 0.197 |
| EYT_1 | E+G+GE | DTMT | Global | 376.15 | 3.243 | 156.038 | 2.669 | 0.415 | 0.823 |
| EYT_1 | E+G+GE | GY | Bed5IR | 0.87 | 2.043 | 0.165 | 0.89 | 0.19 | 0.435 |
| EYT_1 | E+G+GE | GY | EHT | 0.448 | 1.087 | 0.35 | 0.961 | 0.782 | 0.884 |
| EYT_1 | E+G+GE | GY | Flat5IR | 0.369 | 1.016 | 1.229 | 1.854 | 3.331 | 1.825 |
| EYT_1 | E+G+GE | GY | LHT | 9.108 | 7.174 | 5.311 | 5.478 | 0.583 | 0.764 |
| EYT_1 | E+G+GE | GY | Global | 2.699 | 2.83 | 1.764 | 2.296 | 0.654 | 0.811 |
| EYT_1 | E+G+GE | Height | Bed5IR | 155.326 | 2.985 | 122.719 | 2.653 | 0.79 | 0.889 |
| EYT_1 | E+G+GE | Height | EHT | 26.913 | 0.935 | 55.118 | 1.339 | 2.048 | 1.431 |
| EYT_1 | E+G+GE | Height | Flat5IR | 18.411 | 0.959 | 1147.385 | 7.573 | 62.322 | 7.895 |
| EYT_1 | E+G+GE | Height | LHT | 1387.131 | 6.544 | 29.017 | 0.947 | 0.021 | 0.145 |
| EYT_1 | E+G+GE | Height | Global | 396.945 | 2.856 | 338.56 | 3.128 | 0.853 | 1.095 |
| EYT_1 | E+G | DTHD | Bed5IR | 51.633 | 1.083 | 93.332 | 1.457 | 1.808 | 1.344 |
| EYT_1 | E+G | DTHD | EHT | 38.329 | 0.765 | 181.388 | 1.665 | 4.732 | 2.175 |
| EYT_1 | E+G | DTHD | Flat5IR | 16.722 | 1.225 | 13.311 | 1.093 | 0.796 | 0.892 |
| EYT_1 | E+G | DTHD | LHT | 404.607 | 5.553 | 350.142 | 5.166 | 0.865 | 0.93 |
| EYT_1 | E+G | DTHD | Global | 127.823 | 2.157 | 159.544 | 2.345 | 1.248 | 1.087 |
| EYT_1 | E+G | DTMT | Bed5IR | 107.131 | 1.834 | 18.838 | 0.769 | 0.176 | 0.419 |
| EYT_1 | E+G | DTMT | EHT | 117.328 | 1.464 | 1639.806 | 5.474 | 13.976 | 3.738 |
| EYT_1 | E+G | DTMT | Flat5IR | 23.991 | 1.633 | 18.358 | 1.428 | 0.765 | 0.875 |
| EYT_1 | E+G | DTMT | LHT | 1253.278 | 8.175 | 2433.703 | 11.391 | 1.942 | 1.393 |
| EYT_1 | E+G | DTMT | Global | 375.432 | 3.276 | 1027.676 | 4.766 | 2.737 | 1.455 |
| EYT_1 | E+G | GY | Bed5IR | 0.867 | 2.04 | 3.111 | 3.865 | 3.589 | 1.894 |
| EYT_1 | E+G | GY | EHT | 0.464 | 1.106 | 13.378 | 5.939 | 28.858 | 5.372 |
| EYT_1 | E+G | GY | Flat5IR | 0.375 | 1.024 | 2.853 | 2.824 | 7.607 | 2.758 |
| EYT_1 | E+G | GY | LHT | 9.199 | 7.21 | 9.244 | 7.227 | 1.005 | 1.002 |
| EYT_1 | E+G | GY | Global | 2.726 | 2.845 | 7.146 | 4.964 | 2.622 | 1.745 |
| EYT_1 | E+G | Height | Bed5IR | 152.234 | 2.955 | 251.339 | 3.797 | 1.651 | 1.285 |
| EYT_1 | E+G | Height | EHT | 27.997 | 0.954 | 36.585 | 1.091 | 1.307 | 1.143 |
| EYT_1 | E+G | Height | Flat5IR | 18.464 | 0.961 | 86.172 | 2.075 | 4.667 | 2.16 |
| EYT_1 | E+G | Height | LHT | 1402.359 | 6.58 | 778.531 | 4.903 | 0.555 | 0.745 |
| EYT_1 | E+G | Height | Global | 400.264 | 2.862 | 288.157 | 2.966 | 0.72 | 1.036 |

**Appendix B**. Prediction performance for each environment and across environments (Global) of **data set 2** (YET_2) in terms of mean square error (MSE), normalized root mean square error (NRMSE) and relative efficiency (RE) under two predictors (E+G; environments plus genotypes and E+G+GE, that also contain the genotype by environment interaction). MSE_PLS and MSE_G denote the MSE under the PLS and GBLUP method. NRMSE_PLS and NRMSE_G denote the NRMSE under the PLS and GBLUP method. RE_MSE and RE_NRMSE denote the RE computed with the MSE and , respectively. RE were computed by dividing the prediction performance (with MSE or NRMSE) of the GBLUP method by the prediction performance of the PLS method.

| Data | Predictor | Trait | Env | MSE_PLS | NRMSE_PLS | MSE_G | NRMSE_G | RE_MSE | RE_NRMSE |
| --- | --- | --- | --- | --- | --- | --- | --- | --- | --- |
| EYT_2 | E+G+GE | DTHD | Bed2IR | 37.91 | 1.718 | 24.863 | 1.392 | 0.656 | 0.81 |
| EYT_2 | E+G+GE | DTHD | Bed5IR | 9.254 | 0.622 | 17.772 | 0.862 | 1.92 | 1.386 |
| EYT_2 | E+G+GE | DTHD | EHT | 16.603 | 0.689 | 150.515 | 2.074 | 9.066 | 3.011 |
| EYT_2 | E+G+GE | DTHD | Flat5IR | 8.659 | 0.891 | 41.199 | 1.944 | 4.758 | 2.181 |
| EYT_2 | E+G+GE | DTHD | LHT | 496.751 | 8.116 | 557.328 | 8.597 | 1.122 | 1.059 |
| EYT_2 | E+G+GE | DTHD | Global | 113.835 | 2.407 | 158.335 | 2.974 | 1.391 | 1.235 |
| EYT_2 | E+G+GE | DTMT | Bed2IR | 7.222 | 0.81 | 24.72 | 1.498 | 3.423 | 1.85 |
| EYT_2 | E+G+GE | DTMT | Bed5IR | 38.082 | 1.397 | 45.288 | 1.524 | 1.189 | 1.091 |
| EYT_2 | E+G+GE | DTMT | EHT | 200.067 | 2.766 | 257.835 | 3.14 | 1.289 | 1.135 |
| EYT_2 | E+G+GE | DTMT | Flat5IR | 5.864 | 0.674 | 35.697 | 1.663 | 6.087 | 2.467 |
| EYT_2 | E+G+GE | DTMT | LHT | 612.752 | 9.359 | 848.318 | 11.012 | 1.384 | 1.177 |
| EYT_2 | E+G+GE | DTMT | Global | 172.798 | 3.001 | 242.372 | 3.767 | 1.403 | 1.255 |
| EYT_2 | E+G+GE | GY | Bed2IR | 1.332 | 3.161 | 1.85 | 3.724 | 1.388 | 1.178 |
| EYT_2 | E+G+GE | GY | Bed5IR | 3.494 | 4.042 | 2.183 | 3.195 | 0.625 | 0.79 |
| EYT_2 | E+G+GE | GY | EHT | 3.678 | 2.931 | 2.758 | 2.538 | 0.75 | 0.866 |
| EYT_2 | E+G+GE | GY | Flat5IR | 3.333 | 3.571 | 2.551 | 3.124 | 0.765 | 0.875 |
| EYT_2 | E+G+GE | GY | LHT | 1.58 | 2.466 | 2.397 | 3.037 | 1.517 | 1.232 |
| EYT_2 | E+G+GE | GY | Global | 2.683 | 3.234 | 2.348 | 3.124 | 0.875 | 0.966 |
| EYT_2 | E+G+GE | Height | Bed2IR | 27.861 | 0.981 | 320.09 | 3.325 | 11.489 | 3.389 |
| EYT_2 | E+G+GE | Height | Bed5IR | 68.955 | 1.7 | 19.055 | 0.893 | 0.276 | 0.526 |
| EYT_2 | E+G+GE | Height | EHT | 113.841 | 2.077 | 1290.481 | 6.993 | 11.336 | 3.367 |
| EYT_2 | E+G+GE | Height | Flat5IR | 168.536 | 3.255 | 960.187 | 7.769 | 5.697 | 2.387 |
| EYT_2 | E+G+GE | Height | LHT | 439.981 | 4.173 | 114.162 | 2.126 | 0.259 | 0.509 |
| EYT_2 | E+G+GE | Height | Global | 163.835 | 2.437 | 540.795 | 4.222 | 3.301 | 1.732 |
| EYT_2 | E+G | DTHD | Bed2IR | 37.481 | 1.709 | 108.208 | 2.903 | 2.887 | 1.699 |
| EYT_2 | E+G | DTHD | Bed5IR | 6.52 | 0.522 | 17.46 | 0.854 | 2.678 | 1.636 |
| EYT_2 | E+G | DTHD | EHT | 14.262 | 0.638 | 21.364 | 0.781 | 1.498 | 1.224 |
| EYT_2 | E+G | DTHD | Flat5IR | 9.946 | 0.955 | 13.571 | 1.116 | 1.364 | 1.168 |
| EYT_2 | E+G | DTHD | LHT | 496.989 | 8.118 | 732.041 | 9.853 | 1.473 | 1.214 |
| EYT_2 | E+G | DTHD | Global | 113.039 | 2.388 | 178.529 | 3.101 | 1.579 | 1.299 |
| EYT_2 | E+G | DTMT | Bed2IR | 7.089 | 0.802 | 164.471 | 3.865 | 23.2 | 4.817 |
| EYT_2 | E+G | DTMT | Bed5IR | 34.723 | 1.334 | 6.255 | 0.566 | 0.18 | 0.424 |
| EYT_2 | E+G | DTMT | EHT | 196.347 | 2.74 | 399.272 | 3.908 | 2.033 | 1.426 |
| EYT_2 | E+G | DTMT | Flat5IR | 5.71 | 0.665 | 57.658 | 2.113 | 10.099 | 3.178 |
| EYT_2 | E+G | DTMT | LHT | 608.371 | 9.325 | 974.332 | 11.802 | 1.602 | 1.266 |
| EYT_2 | E+G | DTMT | Global | 170.448 | 2.974 | 320.398 | 4.451 | 1.88 | 1.497 |
| EYT_2 | E+G | GY | Bed2IR | 1.361 | 3.194 | 1.181 | 2.976 | 0.868 | 0.931 |
| EYT_2 | E+G | GY | Bed5IR | 3.571 | 4.086 | 0.658 | 1.754 | 0.184 | 0.429 |
| EYT_2 | E+G | GY | EHT | 3.803 | 2.98 | 1.995 | 2.159 | 0.525 | 0.724 |
| EYT_2 | E+G | GY | Flat5IR | 3.506 | 3.663 | 1.142 | 2.09 | 0.326 | 0.571 |
| EYT_2 | E+G | GY | LHT | 1.501 | 2.404 | 13.878 | 7.308 | 9.243 | 3.04 |
| EYT_2 | E+G | GY | Global | 2.748 | 3.265 | 3.771 | 3.257 | 1.372 | 0.998 |
| EYT_2 | E+G | Height | Bed2IR | 28.048 | 0.984 | 51.837 | 1.338 | 1.848 | 1.359 |
| EYT_2 | E+G | Height | Bed5IR | 73.991 | 1.761 | 23.149 | 0.985 | 0.313 | 0.559 |
| EYT_2 | E+G | Height | EHT | 104.999 | 1.995 | 153.572 | 2.413 | 1.463 | 1.209 |
| EYT_2 | E+G | Height | Flat5IR | 167.199 | 3.242 | 1621.096 | 10.095 | 9.696 | 3.114 |
| EYT_2 | E+G | Height | LHT | 433.33 | 4.142 | 724.55 | 5.356 | 1.672 | 1.293 |
| EYT_2 | E+G | Height | Global | 161.513 | 2.425 | 514.841 | 4.037 | 3.188 | 1.665 |

**Appendix C**. Prediction performance for each environment and across environments (Global) of **data set 3** (YET_3) in terms of mean square error (MSE), normalized root mean square error (NRMSE) and relative efficiency (RE) under two predictors (E+G; environments plus genotypes and E+G+GE, that also contain the genotype by environment interaction). MSE_PLS and MSE_G denote the MSE under the PLS and GBLUP method. NRMSE_PLS and NRMSE_G denote the NRMSE under the PLS and GBLUP method. RE_MSE and RE_NRMSE denote the RE computed with the MSE and NRMSE, respectively. RE were computed by dividing the prediction performance (with MSE or NRMSE) of the GBLUP method by the prediction performance of the PLS method.

| Data | Predictor | Trait | Env | MSE_PLS | NRMSE_PLS | MSE_G | NRMSE_G | RE_MSE | RE_NRMSE |
| --- | --- | --- | --- | --- | --- | --- | --- | --- | --- |
| EYT_3 | E+G+GE | DTHD | Bed2IR | 44.306 | 2.192 | 29.025 | 1.775 | 0.655 | 0.809 |
| EYT_3 | E+G+GE | DTHD | Bed5IR | 14.984 | 1.025 | 129.112 | 3.008 | 8.617 | 2.935 |
| EYT_3 | E+G+GE | DTHD | Flat5IR | 4.707 | 0.698 | 88.43 | 3.025 | 18.787 | 4.335 |
| EYT_3 | E+G+GE | DTHD | FlatDrip | 39.937 | 1.384 | 67.441 | 1.799 | 1.689 | 1.299 |
| EYT_3 | E+G+GE | DTHD | LHT | 579.949 | 8.012 | 503.574 | 7.466 | 0.868 | 0.932 |
| EYT_3 | E+G+GE | DTHD | Global | 136.777 | 2.662 | 163.516 | 3.415 | 1.195 | 1.283 |
| EYT_3 | E+G+GE | DTMT | Bed2IR | 13.966 | 1.321 | 55.346 | 2.629 | 3.963 | 1.991 |
| EYT_3 | E+G+GE | DTMT | Bed5IR | 97.091 | 2.754 | 501.433 | 6.258 | 5.165 | 2.273 |
| EYT_3 | E+G+GE | DTMT | Flat5IR | 67.587 | 2.228 | 68.97 | 2.25 | 1.02 | 1.01 |
| EYT_3 | E+G+GE | DTMT | FlatDrip | 31.701 | 1.335 | 20.404 | 1.071 | 0.644 | 0.802 |
| EYT_3 | E+G+GE | DTMT | LHT | 637.079 | 12.776 | 1034.437 | 16.28 | 1.624 | 1.274 |
| EYT_3 | E+G+GE | DTMT | Global | 169.485 | 4.082 | 336.118 | 5.697 | 1.983 | 1.396 |
| EYT_3 | E+G+GE | GY | Bed2IR | 0.759 | 2.794 | 8.697 | 9.455 | 11.455 | 3.385 |
| EYT_3 | E+G+GE | GY | Bed5IR | 8.34 | 7.762 | 16.191 | 10.814 | 1.941 | 1.393 |
| EYT_3 | E+G+GE | GY | Flat5IR | 6.475 | 5.291 | 10.272 | 6.664 | 1.587 | 1.26 |
| EYT_3 | E+G+GE | GY | FlatDrip | 2.255 | 3.229 | 4.699 | 4.662 | 2.084 | 1.444 |
| EYT_3 | E+G+GE | GY | LHT | 0.725 | 2.109 | 4.543 | 5.28 | 6.265 | 2.503 |
| EYT_3 | E+G+GE | GY | Global | 3.711 | 4.237 | 8.881 | 7.375 | 2.393 | 1.741 |
| EYT_3 | E+G+GE | Height | Bed2IR | 86.221 | 1.767 | 73.471 | 1.631 | 0.852 | 0.923 |
| EYT_3 | E+G+GE | Height | Bed5IR | 850.005 | 6.921 | 1207.328 | 8.249 | 1.42 | 1.192 |
| EYT_3 | E+G+GE | Height | Flat5IR | 548.22 | 4.818 | 158.164 | 2.588 | 0.289 | 0.537 |
| EYT_3 | E+G+GE | Height | FlatDrip | 179.783 | 2.406 | 620.753 | 4.471 | 3.453 | 1.858 |
| EYT_3 | E+G+GE | Height | LHT | 87.711 | 2.022 | 385.605 | 4.24 | 4.396 | 2.097 |
| EYT_3 | E+G+GE | Height | Global | 350.388 | 3.587 | 489.064 | 4.236 | 1.396 | 1.181 |
| EYT_3 | E+G | DTHD | Bed2IR | 44.124 | 2.188 | 6.438 | 0.836 | 0.146 | 0.382 |
| EYT_3 | E+G | DTHD | Bed5IR | 13.692 | 0.98 | 226.177 | 3.981 | 16.519 | 4.064 |
| EYT_3 | E+G | DTHD | Flat5IR | 4.793 | 0.704 | 58.277 | 2.456 | 12.158 | 3.487 |
| EYT_3 | E+G | DTHD | FlatDrip | 37.658 | 1.344 | 7.571 | 0.603 | 0.201 | 0.448 |
| EYT_3 | E+G | DTHD | LHT | 579.461 | 8.009 | 741.091 | 9.057 | 1.279 | 1.131 |
| EYT_3 | E+G | DTHD | Global | 135.946 | 2.645 | 207.911 | 3.387 | 1.529 | 1.28 |
| EYT_3 | E+G | DTMT | Bed2IR | 12.86 | 1.267 | 5.4 | 0.821 | 0.42 | 0.648 |
| EYT_3 | E+G | DTMT | Bed5IR | 96.564 | 2.746 | 550.708 | 6.558 | 5.703 | 2.388 |
| EYT_3 | E+G | DTMT | Flat5IR | 66.479 | 2.209 | 226.265 | 4.076 | 3.404 | 1.845 |
| EYT_3 | E+G | DTMT | FlatDrip | 28.467 | 1.265 | 10.741 | 0.777 | 0.377 | 0.614 |
| EYT_3 | E+G | DTMT | LHT | 633.344 | 12.738 | 836.989 | 14.644 | 1.322 | 1.15 |
| EYT_3 | E+G | DTMT | Global | 167.543 | 4.045 | 326.021 | 5.375 | 1.946 | 1.329 |
| EYT_3 | E+G | GY | Bed2IR | 0.753 | 2.782 | 0.225 | 1.521 | 0.299 | 0.547 |
| EYT_3 | E+G | GY | Bed5IR | 8.107 | 7.652 | 6.037 | 6.603 | 0.745 | 0.863 |
| EYT_3 | E+G | GY | Flat5IR | 6.602 | 5.343 | 4.083 | 4.202 | 0.618 | 0.786 |
| EYT_3 | E+G | GY | FlatDrip | 2.18 | 3.175 | 3.165 | 3.826 | 1.452 | 1.205 |
| EYT_3 | E+G | GY | LHT | 0.704 | 2.078 | 0.678 | 2.039 | 0.963 | 0.981 |
| EYT_3 | E+G | GY | Global | 3.669 | 4.206 | 2.837 | 3.638 | 0.773 | 0.865 |
| EYT_3 | E+G | Height | Bed2IR | 87.891 | 1.784 | 73.992 | 1.637 | 0.842 | 0.917 |
| EYT_3 | E+G | Height | Bed5IR | 851.917 | 6.929 | 773.117 | 6.601 | 0.908 | 0.953 |
| EYT_3 | E+G | Height | Flat5IR | 552.386 | 4.836 | 1251.081 | 7.278 | 2.265 | 1.505 |
| EYT_3 | E+G | Height | FlatDrip | 179.162 | 2.402 | 482.389 | 3.942 | 2.692 | 1.641 |
| EYT_3 | E+G | Height | LHT | 86.858 | 2.012 | 269.883 | 3.547 | 3.107 | 1.763 |
| EYT_3 | E+G | Height | Global | 351.643 | 3.593 | 570.092 | 4.601 | 1.621 | 1.281 |

**Appendix D**. Prediction performance for each environment and across environments (Global) of **data set 4** (Groundnut) in terms of mean square error (MSE), normalized root mean square error (NRMSE) and relative efficiency (RE) under two predictors (E+G; environments plus genotypes and E+G+GE, that also contain the genotype by environment interaction). MSE_PLS and MSE_G denote the MSE under the PLS and GBLUP method. NRMSE_PLS and NRMSE_G denote the NRMSE under the PLS and GBLUP method. RE_MSE and RE_NRMSE denote the RE computed with the MSE and NRMSE, respectively. RE were computed by dividing the prediction performance (with MSE or NRMSE) of the GBLUP method by the prediction performance of the PLS method.

| Data | Predictor | Trait | Env | MSE_PLS | NRMSE_PLS | MSE_G | NRMSE_G | RE_MSE | RE_NRMSE |
| --- | --- | --- | --- | --- | --- | --- | --- | --- | --- |
| Groundnut | E+G+GE | NPP | ALIYARNAGAR_R15 | 26.107 | 0.997 | 106.747 | 2.015 | 4.089 | 2.022 |
| Groundnut | E+G+GE | NPP | ICRISAT_PR15-16 | 29.686 | 1.256 | 1076.322 | 7.563 | 36.257 | 6.022 |
| Groundnut | E+G+GE | NPP | ICRISAT_R15 | 18.491 | 0.853 | 63.483 | 1.58 | 3.433 | 1.853 |
| Groundnut | E+G+GE | NPP | JALGOAN_R15 | 33.929 | 0.962 | 342.511 | 3.056 | 10.095 | 3.177 |
| Groundnut | E+G+GE | NPP | Global | 27.053 | 1.017 | 397.266 | 3.553 | 14.685 | 3.495 |
| Groundnut | E+G+GE | PYPP | ALIYARNAGAR_R15 | 18.606 | 1.103 | 48.232 | 1.776 | 2.592 | 1.61 |
| Groundnut | E+G+GE | PYPP | ICRISAT_PR15-16 | 21.388 | 1.598 | 65.406 | 2.795 | 3.058 | 1.749 |
| Groundnut | E+G+GE | PYPP | ICRISAT_R15 | 11.545 | 0.89 | 202.011 | 3.722 | 17.498 | 4.183 |
| Groundnut | E+G+GE | PYPP | JALGOAN_R15 | 15.558 | 0.933 | 26.476 | 1.218 | 1.702 | 1.304 |
| Groundnut | E+G+GE | PYPP | Global | 16.774 | 1.131 | 85.531 | 2.378 | 5.099 | 2.102 |
| Groundnut | E+G+GE | SYPP | ALIYARNAGAR_R15 | 7.399 | 1.12 | 5.977 | 1.006 | 0.808 | 0.899 |
| Groundnut | E+G+GE | SYPP | ICRISAT_PR15-16 | 8.2 | 1.521 | 13.004 | 1.915 | 1.586 | 1.259 |
| Groundnut | E+G+GE | SYPP | ICRISAT_R15 | 4.319 | 0.883 | 276.39 | 7.065 | 63.991 | 8 |
| Groundnut | E+G+GE | SYPP | JALGOAN_R15 | 6.096 | 0.921 | 25.006 | 1.866 | 4.102 | 2.025 |
| Groundnut | E+G+GE | SYPP | Global | 6.503 | 1.111 | 80.094 | 2.963 | 12.316 | 2.666 |
| Groundnut | E+G+GE | YPH | ALIYARNAGAR_R15 | 365664.2 | 0.887 | 1106988 | 1.543 | 3.027 | 1.74 |
| Groundnut | E+G+GE | YPH | ICRISAT_PR15-16 | 556877.7 | 0.986 | 10512652 | 4.284 | 18.878 | 4.345 |
| Groundnut | E+G+GE | YPH | ICRISAT_R15 | 291177.9 | 0.796 | 251066.1 | 0.739 | 0.862 | 0.929 |
| Groundnut | E+G+GE | YPH | JALGOAN_R15 | 454937.8 | 0.844 | 4409167 | 2.626 | 9.692 | 3.113 |
| Groundnut | E+G+GE | YPH | Global | 417164.4 | 0.878 | 4069968 | 2.298 | 9.756 | 2.617 |
| Groundnut | E+G | NPP | ALIYARNAGAR_R15 | 26.927 | 1.012 | 25.059 | 0.976 | 0.931 | 0.965 |
| Groundnut | E+G | NPP | ICRISAT_PR15-16 | 35.614 | 1.376 | 32.626 | 1.317 | 0.916 | 0.957 |
| Groundnut | E+G | NPP | ICRISAT_R15 | 17.446 | 0.828 | 448.703 | 4.199 | 25.719 | 5.072 |
| Groundnut | E+G | NPP | JALGOAN_R15 | 30.428 | 0.911 | 189.232 | 2.271 | 6.219 | 2.494 |
| Groundnut | E+G | NPP | Global | 27.604 | 1.032 | 173.905 | 2.191 | 6.3 | 2.124 |
| Groundnut | E+G | PYPP | ALIYARNAGAR_R15 | 19.225 | 1.121 | 15.351 | 1.002 | 0.799 | 0.894 |
| Groundnut | E+G | PYPP | ICRISAT_PR15-16 | 29.726 | 1.884 | 322.005 | 6.202 | 10.832 | 3.291 |
| Groundnut | E+G | PYPP | ICRISAT_R15 | 10.977 | 0.868 | 48.269 | 1.819 | 4.397 | 2.097 |
| Groundnut | E+G | PYPP | JALGOAN_R15 | 16.217 | 0.953 | 64.516 | 1.901 | 3.978 | 1.995 |
| Groundnut | E+G | PYPP | Global | 19.036 | 1.207 | 112.535 | 2.731 | 5.912 | 2.263 |
| Groundnut | E+G | SYPP | ALIYARNAGAR_R15 | 7.264 | 1.109 | 14.244 | 1.554 | 1.961 | 1.4 |
| Groundnut | E+G | SYPP | ICRISAT_PR15-16 | 10.851 | 1.749 | 149.807 | 6.5 | 13.806 | 3.716 |
| Groundnut | E+G | SYPP | ICRISAT_R15 | 4.82 | 0.933 | 283.316 | 7.153 | 58.778 | 7.667 |
| Groundnut | E+G | SYPP | JALGOAN_R15 | 6.608 | 0.959 | 142.713 | 4.458 | 21.596 | 4.647 |
| Groundnut | E+G | SYPP | Global | 7.386 | 1.188 | 147.52 | 4.916 | 19.973 | 4.139 |
| Groundnut | E+G | YPH | ALIYARNAGAR_R15 | 453699 | 0.988 | 5134421 | 3.322 | 11.317 | 3.364 |
| Groundnut | E+G | YPH | ICRISAT_PR15-16 | 718906.9 | 1.12 | 2270725 | 1.991 | 3.159 | 1.777 |
| Groundnut | E+G | YPH | ICRISAT_R15 | 305708.3 | 0.815 | 1998757 | 2.084 | 6.538 | 2.557 |
| Groundnut | E+G | YPH | JALGOAN_R15 | 468764.7 | 0.856 | 2443929 | 1.955 | 5.214 | 2.283 |
| Groundnut | E+G | YPH | Global | 486769.7 | 0.945 | 2961958 | 2.338 | 6.085 | 2.475 |

**Appendix E**. Prediction performance for each environment and across environments (Global) of **data set 5** (Maize) in terms of mean square error (MSE), normalized root mean square error (NRMSE) and relative efficiency (RE) under two predictors (E+G; environments plus genotypes and E+G+GE, that also contain the genotype by environment interaction). MSE_PLS and MSE_G denote the MSE under the PLS and GBLUP method. NRMSE_PLS and NRMSE_G denote the NRMSE under the PLS and GBLUP method. RE_MSE and RE_NRMSE denote the RE computed with the MSE and NRMSE, respectively. RE were computed by dividing the prediction performance (with MSE or NRMSE) of the GBLUP method by the prediction performance of the PLS method.

| Data | Predictor | Trait | Env | MSE_PLS | NRMSE_PLS | MSE_G | NRMSE_G | RE_MSE | RE_NRMSE |
| --- | --- | --- | --- | --- | --- | --- | --- | --- | --- |
| Maize | E+G+GE | GY | Env1 | 0.109 | 1.129 | 0.118 | 1.173 | 1.079 | 1.038 |
| Maize | E+G+GE | GY | Env2 | 0.25 | 0.825 | 2.918 | 2.82 | 11.674 | 3.417 |
| Maize | E+G+GE | GY | Env3 | 0.131 | 1.019 | 4.114 | 5.714 | 31.474 | 5.611 |
| Maize | E+G+GE | GY | Env4 | 0.415 | 0.845 | 3.176 | 2.338 | 7.657 | 2.767 |
| Maize | E+G+GE | GY | Global | 0.226 | 0.955 | 2.581 | 3.011 | 11.417 | 3.155 |
| Maize | E+G | GY | Env1 | 0.152 | 1.332 | 0.097 | 1.067 | 0.642 | 0.801 |
| Maize | E+G | GY | Env2 | 0.332 | 0.951 | 0.36 | 0.99 | 1.084 | 1.041 |
| Maize | E+G | GY | Env3 | 0.167 | 1.15 | 0.122 | 0.982 | 0.73 | 0.854 |
| Maize | E+G | GY | Env4 | 0.395 | 0.825 | 3.034 | 2.285 | 7.681 | 2.772 |
| Maize | E+G | GY | Global | 0.261 | 1.064 | 0.903 | 1.331 | 3.458 | 1.251 |

**Appendix F**. Prediction performance for each environment and across environments (Global) of **data set 6** (Disease) in terms of mean square error (MSE), normalized root mean square error (NRMSE) and relative efficiency (RE) under two predictors (E+G; environments plus genotypes and E+G+GE, that also contain the genotype by environment interaction). MSE_PLS and MSE_G denotes the MSE under the PLS and GBLUP method. NRMSE_PLS and NRMSE_G denotes the NRMSE under the PLS and GBLUP method. RE_MSE and RE_NRMSE denotes the RE computed with the MSE and NRMSE respectively. RE were computed dividing the prediction performance (with MSE or NRMSE) of the GBLUP method by the prediction performance of the PLS method.

| Data | Predictor | Trait | Env | MSE_PLS | NRMSE_PLS | MSE_G | NRMSE_G | RE_MSE | RE_NRMSE |
| --- | --- | --- | --- | --- | --- | --- | --- | --- | --- |
| Disease | E+G+GE | SN | Env1 | 12.894 | 0.78 | 21.19 | 1 | 1.643 | 1.282 |
| Disease | E+G+GE | SN | Env2 | 11.201 | 0.759 | 798.88 | 6.407 | 71.324 | 8.445 |
| Disease | E+G+GE | SN | Env3 | 8.559 | 0.864 | 100.768 | 2.964 | 11.774 | 3.431 |
| Disease | E+G+GE | SN | Env4 | 6.653 | 0.762 | 10.934 | 0.977 | 1.643 | 1.282 |
| Disease | E+G+GE | SN | Env5 | 7.594 | 0.838 | 241.794 | 4.731 | 31.841 | 5.643 |
| Disease | E+G+GE | SN | Env6 | 7.436 | 0.784 | 12.411 | 1.013 | 1.669 | 1.292 |
| Disease | E+G+GE | SN | Global | 9.056 | 0.798 | 197.663 | 2.849 | 21.826 | 3.57 |
| Disease | E+G+GE | PTR | Env1 | 13.909 | 0.984 | 181.8 | 3.557 | 13.071 | 3.615 |
| Disease | E+G+GE | PTR | Env2 | 11.675 | 0.904 | 15.623 | 1.045 | 1.338 | 1.157 |
| Disease | E+G+GE | PTR | Env3 | 15.582 | 0.983 | 13.546 | 0.917 | 0.869 | 0.932 |
| Disease | E+G+GE | PTR | Env4 | 14.82 | 0.966 | 13.404 | 0.918 | 0.904 | 0.951 |
| Disease | E+G+GE | PTR | Env5 | 13.537 | 1.114 | 8.825 | 0.899 | 0.652 | 0.807 |
| Disease | E+G+GE | PTR | Env6 | 12.574 | 1.142 | 97.73 | 3.182 | 7.773 | 2.788 |
| Disease | E+G+GE | PTR | Global | 13.683 | 1.015 | 55.155 | 1.753 | 4.031 | 1.727 |
| Disease | E+G+GE | SB | Env1 | 7.439 | 0.861 | 132.553 | 3.632 | 17.818 | 4.221 |
| Disease | E+G+GE | SB | Env2 | 6.418 | 0.874 | 259.571 | 5.555 | 40.442 | 6.36 |
| Disease | E+G+GE | SB | Env3 | 6.833 | 0.843 | 287.878 | 5.47 | 42.131 | 6.491 |
| Disease | E+G+GE | SB | Env4 | 7.082 | 0.86 | 13.645 | 1.194 | 1.927 | 1.388 |
| Disease | E+G+GE | SB | Env5 | 7.809 | 0.838 | 33.712 | 1.741 | 4.317 | 2.078 |
| Disease | E+G+GE | SB | Env6 | 8.386 | 0.866 | 19.029 | 1.305 | 2.269 | 1.506 |
| Disease | E+G+GE | SB | Global | 7.328 | 0.857 | 124.398 | 3.149 | 16.976 | 3.676 |
| Disease | E+G | SN | Env1 | 12.536 | 0.769 | 102.544 | 2.201 | 8.18 | 2.86 |
| Disease | E+G | SN | Env2 | 10.906 | 0.749 | 11.272 | 0.761 | 1.034 | 1.017 |
| Disease | E+G | SN | Env3 | 8.556 | 0.864 | 120.173 | 3.236 | 14.045 | 3.748 |
| Disease | E+G | SN | Env4 | 6.643 | 0.762 | 546.655 | 6.911 | 82.292 | 9.072 |
| Disease | E+G | SN | Env5 | 8.297 | 0.876 | 120.955 | 3.346 | 14.578 | 3.818 |
| Disease | E+G | SN | Env6 | 7.8 | 0.803 | 129.579 | 3.272 | 16.613 | 4.076 |
| Disease | E+G | SN | Global | 9.123 | 0.804 | 171.863 | 3.288 | 18.838 | 4.09 |
| Disease | E+G | PTR | Env1 | 11.542 | 0.896 | 281.153 | 4.423 | 24.359 | 4.936 |
| Disease | E+G | PTR | Env2 | 11.797 | 0.908 | 15.324 | 1.035 | 1.299 | 1.14 |
| Disease | E+G | PTR | Env3 | 11.688 | 0.852 | 194.516 | 3.474 | 16.643 | 4.08 |
| Disease | E+G | PTR | Env4 | 11.995 | 0.869 | 17.101 | 1.037 | 1.426 | 1.194 |
| Disease | E+G | PTR | Env5 | 11.207 | 1.014 | 19.389 | 1.333 | 1.73 | 1.315 |
| Disease | E+G | PTR | Env6 | 11.053 | 1.07 | 534.235 | 7.441 | 48.334 | 6.952 |
| Disease | E+G | PTR | Global | 11.547 | 0.935 | 176.953 | 3.124 | 15.325 | 3.342 |
| Disease | E+G | SB | Env1 | 7.329 | 0.854 | 35.151 | 1.871 | 4.796 | 2.19 |
| Disease | E+G | SB | Env2 | 6.61 | 0.887 | 32.398 | 1.963 | 4.901 | 2.214 |
| Disease | E+G | SB | Env3 | 6.861 | 0.844 | 45.178 | 2.167 | 6.585 | 2.566 |
| Disease | E+G | SB | Env4 | 7.661 | 0.895 | 249.215 | 5.102 | 32.529 | 5.703 |
| Disease | E+G | SB | Env5 | 7.724 | 0.833 | 319.754 | 5.362 | 41.398 | 6.434 |
| Disease | E+G | SB | Env6 | 8.378 | 0.866 | 10.59 | 0.973 | 1.264 | 1.124 |
| Disease | E+G | SB | Global | 7.427 | 0.863 | 115.381 | 2.906 | 15.535 | 3.367 |

**Appendix G**. Prediction performance for each environment and across environments (Global) of **data set 7-12** (Wheat_1,…, Wheat_6) in terms of mean square error (MSE), normalized root mean square error (NRMSE) and relative efficiency (RE) under two predictors (E+G; environments plus genotypes and E+G+GE, that also contain the genotype by environment interaction). MSE_PLS and MSE_G denote the MSE under the PLS and GBLUP method. NRMSE_PLS and NRMSE_G denote the NRMSE under the PLS and GBLUP method. RE_MSE and RE_NRMSE denote the RE computed with the MSE and NRMSE, respectively. RE were computed by dividing the prediction performance (with MSE or NRMSE) of the GBLUP method by the prediction performance of the PLS method.

| Data | Predictor | Trait | Env | MSE_PLS | NRMSE_PLS | MSE_G | NRMSE_G | RE_MSE | RE_NRMSE |
| --- | --- | --- | --- | --- | --- | --- | --- | --- | --- |
| Wheat_1 | E+G | GY | YT_13_14 | 2.584 | 3.796 | 3.655 | 4.515 | 1.414 | 1.189 |
| Wheat_1 | E+G | GY | YT_14_15 | 2.583 | 3.68 | 11.626 | 7.807 | 4.501 | 2.122 |
| Wheat_1 | E+G | GY | Global | 2.583 | 3.738 | 7.641 | 6.161 | 2.958 | 1.648 |
| Wheat_1 | E+G+GE | GY | YT_13_14 | 3.014 | 4.1 | 2.526 | 3.754 | 0.838 | 0.915 |
| Wheat_1 | E+G+GE | GY | YT_14_15 | 2.057 | 3.284 | 2.95 | 3.932 | 1.434 | 1.198 |
| Wheat_1 | E+G+GE | GY | Global | 2.536 | 3.692 | 2.738 | 3.843 | 1.08 | 1.041 |
| Wheat_2 | E+G | GY | YT_14_15 | 0.794 | 3.093 | 5.218 | 7.928 | 6.571 | 2.563 |
| Wheat_2 | E+G | GY | YT_15_16 | 0.84 | 2.377 | 0.142 | 0.977 | 0.169 | 0.411 |
| Wheat_2 | E+G | GY | Global | 0.817 | 2.735 | 2.68 | 4.452 | 3.279 | 1.628 |
| Wheat_2 | E+G+GE | GY | YT_14_15 | 0.895 | 3.284 | 1.407 | 4.117 | 1.572 | 1.254 |
| Wheat_2 | E+G+GE | GY | YT_15_16 | 0.977 | 2.563 | 3.433 | 4.805 | 3.514 | 1.875 |
| Wheat_2 | E+G+GE | GY | Global | 0.936 | 2.923 | 2.42 | 4.461 | 2.586 | 1.526 |
| Wheat_3 | E+G | GY | YT_15_16 | 1.394 | 4.006 | 3.116 | 5.989 | 2.235 | 1.495 |
| Wheat_3 | E+G | GY | YT_16_17 | 1.498 | 2.381 | 1.121 | 2.06 | 0.748 | 0.865 |
| Wheat_3 | E+G | GY | Global | 1.446 | 3.194 | 2.118 | 4.025 | 1.465 | 1.26 |
| Wheat_3 | E+G+GE | GY | YT_15_16 | 1.926 | 4.709 | 3.675 | 6.505 | 1.908 | 1.381 |
| Wheat_3 | E+G+GE | GY | YT_16_17 | 0.809 | 1.749 | 2 | 2.751 | 2.473 | 1.573 |
| Wheat_3 | E+G+GE | GY | Global | 1.367 | 3.229 | 2.837 | 4.628 | 2.075 | 1.433 |
| Wheat_4 | E+G | GY | YT_16_17 | 0.161 | 1.128 | 0.68 | 2.319 | 4.22 | 2.055 |
| Wheat_4 | E+G | GY | YT_17_18 | 0.216 | 1.124 | 1.14 | 2.579 | 5.27 | 2.296 |
| Wheat_4 | E+G | GY | Global | 0.189 | 1.126 | 0.91 | 2.449 | 4.822 | 2.175 |
| Wheat_4 | E+G+GE | GY | YT_16_17 | 0.17 | 1.159 | 0.986 | 2.793 | 5.807 | 2.41 |
| Wheat_4 | E+G+GE | GY | YT_17_18 | 0.171 | 1 | 4.409 | 5.072 | 25.74 | 5.073 |
| Wheat_4 | E+G+GE | GY | Global | 0.171 | 1.079 | 2.698 | 3.933 | 15.813 | 3.643 |
| Wheat_5 | E+G | GY | YT_17_18 | 0.167 | 1.251 | 0.401 | 1.939 | 2.404 | 1.55 |
| Wheat_5 | E+G | GY | YT_18_19 | 0.245 | 1 | 1.752 | 2.677 | 7.164 | 2.677 |
| Wheat_5 | E+G | GY | Global | 0.206 | 1.125 | 1.077 | 2.308 | 5.233 | 2.051 |
| Wheat_5 | E+G+GE | GY | YT_17_18 | 0.119 | 1.056 | 1.002 | 3.063 | 8.41 | 2.9 |
| Wheat_5 | E+G+GE | GY | YT_18_19 | 0.233 | 0.976 | 0.249 | 1.008 | 1.066 | 1.033 |
| Wheat_5 | E+G+GE | GY | Global | 0.176 | 1.016 | 0.625 | 2.036 | 3.55 | 2.003 |
| Wheat_6 | E+G | GY | YT_18_19 | 5.362 | 5.752 | 1.723 | 3.261 | 0.321 | 0.567 |
| Wheat_6 | E+G | GY | YT_19_20 | 5.136 | 6.393 | 0.173 | 1.174 | 0.034 | 0.184 |
| Wheat_6 | E+G | GY | Global | 5.249 | 6.073 | 0.948 | 2.217 | 0.181 | 0.365 |
| Wheat_6 | E+G+GE | GY | YT_18_19 | 5.156 | 5.641 | 2.018 | 3.529 | 0.391 | 0.626 |
| Wheat_6 | E+G+GE | GY | YT_19_20 | 5.201 | 6.434 | 2.958 | 4.852 | 0.569 | 0.754 |
| Wheat_6 | E+G+GE | GY | Global | 5.178 | 6.037 | 2.488 | 4.19 | 0.48 | 0.694 |

**Appendix H**. Prediction performance for each environment and across environments (Global) of **data set 13** (Indica) in terms of mean square error (MSE), normalized root mean square error (NRMSE) and relative efficiency (RE) under two predictors (E+G; environments plus genotypes and E+G+GE, that also contain the genotype by environment interaction). MSE_PLS and MSE_G denote the MSE under the PLS and GBLUP method. NRMSE_PLS and NRMSE_G denote the NRMSE under the PLS and GBLUP method. RE_MSE and RE_NRMSE denote the RE computed with the MSE and NRMSE, respectively. RE were computed by dividing the prediction performance (with MSE or NRMSE) of the GBLUP method by the prediction performance of the PLS method. EC denotes environment covariates with YES and NO.

| Data | Predictor | EC | Trait | Env | MSE_G | NRMSE_G | MSE_P | NRMSE_P | RE_MSE | RE_NRMSE |
| --- | --- | --- | --- | --- | --- | --- | --- | --- | --- | --- |
| Indica | E+G | YES | GY | 2010 | 972022.759 | 1.181 | 996077.853 | 1.195 | 0.976 | 0.988 |
| Indica | E+G | YES | GY | 2011 | 1563619.334 | 1.270 | 1625892.826 | 1.295 | 0.962 | 0.981 |
| Indica | E+G | YES | GY | 2012 | 2721789.334 | 1.301 | 2664099.650 | 1.287 | 1.022 | 1.011 |
| Indica | E+G | YES | GY | Global | 1752477.142 | 1.250 | 1762023.443 | 1.259 | 0.995 | 0.993 |
| Indica | E+G | YES | PHR | 2010 | 5.117 | 0.946 | 6.158 | 1.038 | 0.831 | 0.911 |
| Indica | E+G | YES | PHR | 2011 | 5.747 | 0.937 | 6.618 | 1.005 | 0.868 | 0.932 |
| Indica | E+G | YES | PHR | 2012 | 10.467 | 0.868 | 11.487 | 0.909 | 0.911 | 0.955 |
| Indica | E+G | YES | PHR | Global | 7.110 | 0.917 | 8.088 | 0.984 | 0.879 | 0.932 |
| Indica | E+G | YES | GC | 2010 | 3.084 | 1.082 | 3.192 | 1.101 | 0.966 | 0.983 |
| Indica | E+G | YES | GC | 2011 | 5.915 | 0.929 | 6.792 | 0.995 | 0.871 | 0.933 |
| Indica | E+G | YES | GC | 2012 | 5.010 | 0.912 | 5.151 | 0.924 | 0.973 | 0.986 |
| Indica | E+G | YES | GC | Global | 4.670 | 0.974 | 5.045 | 1.007 | 0.926 | 0.968 |
| Indica | E+G | YES | PH | 2010 | 6.802 | 0.780 | 6.865 | 0.784 | 0.991 | 0.995 |
| Indica | E+G | YES | PH | 2011 | 19.978 | 1.429 | 20.987 | 1.465 | 0.952 | 0.976 |
| Indica | E+G | YES | PH | 2012 | 25.077 | 1.076 | 24.414 | 1.062 | 1.027 | 1.013 |
| Indica | E+G | YES | PH | Global | 17.285 | 1.095 | 17.422 | 1.104 | 0.992 | 0.992 |
| Indica | E+G+GE | YES | GY | 2010 | 669718.208 | 0.980 | 849755.139 | 1.104 | 0.788 | 0.888 |
| Indica | E+G+GE | YES | GY | 2011 | 1705284.913 | 1.326 | 1672440.481 | 1.314 | 1.020 | 1.010 |
| Indica | E+G+GE | YES | GY | 2012 | 2771914.768 | 1.312 | 2718076.888 | 1.300 | 1.020 | 1.010 |
| Indica | E+G+GE | YES | GY | Global | 1715639.296 | 1.206 | 1746757.503 | 1.239 | 0.982 | 0.974 |
| Indica | E+G+GE | YES | PHR | 2010 | 4.827 | 0.919 | 5.188 | 0.953 | 0.930 | 0.965 |
| Indica | E+G+GE | YES | PHR | 2011 | 5.463 | 0.913 | 6.097 | 0.965 | 0.896 | 0.947 |
| Indica | E+G+GE | YES | PHR | 2012 | 10.514 | 0.870 | 11.698 | 0.918 | 0.899 | 0.948 |
| Indica | E+G+GE | YES | PHR | Global | 6.935 | 0.901 | 7.661 | 0.945 | 0.905 | 0.953 |
| Indica | E+G+GE | YES | GC | 2010 | 2.829 | 1.037 | 2.984 | 1.065 | 0.948 | 0.974 |
| Indica | E+G+GE | YES | GC | 2011 | 5.927 | 0.930 | 6.793 | 0.995 | 0.872 | 0.934 |
| Indica | E+G+GE | YES | GC | 2012 | 5.060 | 0.916 | 5.144 | 0.924 | 0.984 | 0.992 |
| Indica | E+G+GE | YES | GC | Global | 4.605 | 0.961 | 4.974 | 0.995 | 0.926 | 0.966 |
| Indica | E+G+GE | YES | PH | 2010 | 7.802 | 0.836 | 7.334 | 0.810 | 1.064 | 1.031 |
| Indica | E+G+GE | YES | PH | 2011 | 20.221 | 1.438 | 19.425 | 1.409 | 1.041 | 1.020 |
| Indica | E+G+GE | YES | PH | 2012 | 23.096 | 1.033 | 26.967 | 1.116 | 0.856 | 0.925 |
| Indica | E+G+GE | YES | PH | Global | 17.040 | 1.102 | 17.909 | 1.112 | 0.951 | 0.991 |
| Indica | E+G | NO | GY | 2010 | 919216.429 | 1.148 | 1008581.627 | 1.203 | 0.911 | 0.955 |
| Indica | E+G | NO | GY | 2011 | 1549737.933 | 1.265 | 1615184.014 | 1.291 | 0.959 | 0.980 |
| Indica | E+G | NO | GY | 2012 | 2474993.081 | 1.240 | 2420602.702 | 1.227 | 1.022 | 1.011 |
| Indica | E+G | NO | GY | Global | 1647982.481 | 1.218 | 1681456.114 | 1.240 | 0.980 | 0.982 |
| Indica | E+G | NO | PHR | 2010 | 5.117 | 0.946 | 6.132 | 1.036 | 0.834 | 0.913 |
| Indica | E+G | NO | PHR | 2011 | 5.750 | 0.937 | 6.611 | 1.005 | 0.870 | 0.933 |
| Indica | E+G | NO | PHR | 2012 | 10.485 | 0.869 | 11.511 | 0.910 | 0.911 | 0.954 |
| Indica | E+G | NO | PHR | Global | 7.117 | 0.917 | 8.085 | 0.984 | 0.880 | 0.933 |
| Indica | E+G | NO | GC | 2010 | 3.083 | 1.082 | 3.164 | 1.096 | 0.974 | 0.987 |
| Indica | E+G | NO | GC | 2011 | 5.915 | 0.929 | 6.764 | 0.993 | 0.874 | 0.935 |
| Indica | E+G | NO | GC | 2012 | 5.026 | 0.913 | 5.171 | 0.926 | 0.972 | 0.986 |
| Indica | E+G | NO | GC | Global | 4.674 | 0.975 | 5.033 | 1.005 | 0.929 | 0.970 |
| Indica | E+G | NO | PH | 2010 | 4.806 | 0.656 | 6.183 | 0.744 | 0.777 | 0.882 |
| Indica | E+G | NO | PH | 2011 | 20.596 | 1.451 | 20.889 | 1.462 | 0.986 | 0.993 |
| Indica | E+G | NO | PH | 2012 | 22.535 | 1.020 | 21.404 | 0.994 | 1.053 | 1.026 |
| Indica | E+G | NO | PH | Global | 15.979 | 1.043 | 16.159 | 1.067 | 0.989 | 0.977 |
| Indica | E+G+GE | NO | GY | 2010 | 734514.100 | 1.026 | 914257.169 | 1.145 | 0.803 | 0.896 |
| Indica | E+G+GE | NO | GY | 2011 | 1653481.460 | 1.306 | 1652444.763 | 1.306 | 1.001 | 1.000 |
| Indica | E+G+GE | NO | GY | 2012 | 2712673.605 | 1.298 | 2524032.983 | 1.252 | 1.075 | 1.037 |
| Indica | E+G+GE | NO | GY | Global | 1700223.055 | 1.210 | 1696911.638 | 1.234 | 1.002 | 0.980 |
| Indica | E+G+GE | NO | PHR | 2010 | 5.023 | 0.938 | 5.322 | 0.965 | 0.944 | 0.972 |
| Indica | E+G+GE | NO | PHR | 2011 | 5.419 | 0.910 | 6.049 | 0.961 | 0.896 | 0.947 |
| Indica | E+G+GE | NO | PHR | 2012 | 10.315 | 0.862 | 11.672 | 0.916 | 0.884 | 0.940 |
| Indica | E+G+GE | NO | PHR | Global | 6.919 | 0.903 | 7.681 | 0.948 | 0.901 | 0.953 |
| Indica | E+G+GE | NO | GC | 2010 | 2.872 | 1.044 | 2.993 | 1.066 | 0.960 | 0.979 |
| Indica | E+G+GE | NO | GC | 2011 | 6.082 | 0.942 | 6.753 | 0.992 | 0.901 | 0.949 |
| Indica | E+G+GE | NO | GC | 2012 | 5.090 | 0.919 | 5.174 | 0.927 | 0.984 | 0.992 |
| Indica | E+G+GE | NO | GC | Global | 4.681 | 0.968 | 4.973 | 0.995 | 0.941 | 0.973 |
| Indica | E+G+GE | NO | PH | 2010 | 5.233 | 0.684 | 6.251 | 0.748 | 0.837 | 0.915 |
| Indica | E+G+GE | NO | PH | 2011 |  |  |  |  |  |  |
| Indica | E+G+GE | NO | PH | 2012 | 22.112 | 1.011 | 22.506 | 1.020 | 0.982 | 0.991 |
| Indica | E+G+GE | NO | PH | Global | 15.539 | 1.033 | 15.943 | 1.055 | 0.975 | 0.979 |

**Appendix I**. Prediction performance for each environment and across environments (Global) of **data set 14** (Japonica) in terms of mean square error (MSE), normalized root mean square error (NRMSE) and relative efficiency (RE) under two predictors (E+G; environments plus genotypes and E+G+GE, that also contain the genotype by environment interaction). MSE_PLS and MSE_G denote the MSE under the PLS and GBLUP method. NRMSE_PLS and NRMSE_G denote the NRMSE under the PLS and GBLUP method. RE_MSE and RE_NRMSE denote the RE computed with the MSE and NRMSE, respectively. RE were computed by dividing the prediction performance (with MSE or NRMSE) of the GBLUP method by the prediction performance of the PLS method. EC denotes environment covariates with YES and NO.

| Data | Predictor | EC | Trait | Env | MSE_G | NRMSE_G | MSE_P | NRMSE_P | RE_MSE | RE_NRMSE |
| --- | --- | --- | --- | --- | --- | --- | --- | --- | --- | --- |
| Japonica | E+G | YES | GY | 2009 | 1012351.1 | 1.3 | 758119.5 | 1.141 | 1.335 | 1.156 |
| Japonica | E+G | YES | GY | 2010 | 4378560.1 | 2.1 | 3646762.0 | 1.945 | 1.201 | 1.096 |
| Japonica | E+G | YES | GY | 2011 | 2939750.0 | 2.0 | 3405161.8 | 2.161 | 0.863 | 0.929 |
| Japonica | E+G | YES | GY | 2012 | 999247.9 | 1.3 | 1422524.0 | 1.538 | 0.702 | 0.838 |
| Japonica | E+G | YES | GY | 2013 | 355008.6 | 0.9 | 389836.7 | 0.895 | 0.911 | 0.954 |
| Japonica | E+G | YES | GY | Global | 1936983.5 | 1.5 | 1924480.8 | 1.536 | 1.006 | 0.990 |
| Japonica | E+G | YES | PHR | 2009 | 0.0 | 0.8 | 0.0 | 0.900 | 0.750 | 0.888 |
| Japonica | E+G | YES | PHR | 2010 | 0.0 | 1.0 | 0.0 | 1.404 | 0.462 | 0.684 |
| Japonica | E+G | YES | PHR | 2011 | 0.0 | 1.2 | 0.0 | 1.337 | 0.857 | 0.914 |
| Japonica | E+G | YES | PHR | 2012 | 0.0 | 2.6 | 0.0 | 2.631 | 0.967 | 0.985 |
| Japonica | E+G | YES | PHR | 2013 | 0.0 | 1.0 | 0.0 | 0.928 | 1.167 | 1.064 |
| Japonica | E+G | YES | PHR | Global | 0.0 | 1.3 | 0.0 | 1.440 | 0.900 | 0.911 |
| Japonica | E+G | YES | GC | 2009 | 0.0 | 6.7 | 0.0 | 6.117 | 1.182 | 1.089 |
| Japonica | E+G | YES | GC | 2010 | 0.0 | 10.4 | 0.0 | 7.802 | 1.779 | 1.332 |
| Japonica | E+G | YES | GC | 2011 | 0.0 | 1.0 | 0.0 | 0.911 | 1.300 | 1.136 |
| Japonica | E+G | YES | GC | 2012 | 0.0 | 3.4 | 0.0 | 3.245 | 1.094 | 1.047 |
| Japonica | E+G | YES | GC | 2013 | 0.0 | 1.9 | 0.0 | 1.122 | 2.800 | 1.690 |
| Japonica | E+G | YES | GC | Global | 0.0 | 4.7 | 0.0 | 3.840 | 1.386 | 1.218 |
| Japonica | E+G | YES | PH | 2009 | 15.2 | 0.5 | 16.5 | 0.550 | 0.920 | 0.959 |
| Japonica | E+G | YES | PH | 2010 | 23.0 | 0.9 | 13.6 | 0.656 | 1.694 | 1.302 |
| Japonica | E+G | YES | PH | 2011 | 26.2 | 1.0 | 31.0 | 1.082 | 0.844 | 0.919 |
| Japonica | E+G | YES | PH | 2012 | 147.6 | 2.4 | 149.7 | 2.368 | 0.986 | 0.993 |
| Japonica | E+G | YES | PH | 2013 | 15.8 | 0.7 | 28.3 | 0.885 | 0.556 | 0.746 |
| Japonica | E+G | YES | PH | Global | 45.5 | 1.1 | 47.8 | 1.108 | 0.952 | 0.972 |
| Japonica | E+G+GE | YES | GY | 2009 | 628054.1 | 1.0 | 775999.7 | 1.154 | 0.809 | 0.900 |
| Japonica | E+G+GE | YES | GY | 2010 | 3729571.2 | 2.0 | 3639976.2 | 1.944 | 1.025 | 1.012 |
| Japonica | E+G+GE | YES | GY | 2011 | 3865840.0 | 2.3 | 3415139.3 | 2.164 | 1.132 | 1.064 |
| Japonica | E+G+GE | YES | GY | 2012 | 1630068.8 | 1.6 | 1420912.1 | 1.537 | 1.147 | 1.071 |
| Japonica | E+G+GE | YES | GY | 2013 | 384024.7 | 0.9 | 419659.6 | 0.929 | 0.915 | 0.957 |
| Japonica | E+G+GE | YES | GY | Global | 2047511.8 | 1.6 | 1934337.4 | 1.545 | 1.059 | 1.015 |
| Japonica | E+G+GE | YES | PHR | 2009 | 0.0 | 0.9 | 0.0 | 0.812 | 1.000 | 1.065 |
| Japonica | E+G+GE | YES | PHR | 2010 | 0.0 | 1.8 | 0.0 | 1.415 | 1.538 | 1.240 |
| Japonica | E+G+GE | YES | PHR | 2011 | 0.0 | 1.0 | 0.0 | 1.340 | 0.500 | 0.709 |
| Japonica | E+G+GE | YES | PHR | 2012 | 0.0 | 2.7 | 0.0 | 2.627 | 1.034 | 1.015 |
| Japonica | E+G+GE | YES | PHR | 2013 | 0.0 | 0.9 | 0.0 | 0.914 | 1.000 | 0.991 |
| Japonica | E+G+GE | YES | PHR | Global | 0.0 | 1.4 | 0.0 | 1.422 | 1.000 | 1.005 |
| Japonica | E+G+GE | YES | GC | 2009 | 0.0 | 5.1 | 0.0 | 6.120 | 0.688 | 0.831 |
| Japonica | E+G+GE | YES | GC | 2010 | 0.0 | 8.3 | 0.0 | 8.030 | 1.081 | 1.039 |
| Japonica | E+G+GE | YES | GC | 2011 | 0.0 | 0.9 | 0.0 | 0.904 | 1.000 | 1.004 |
| Japonica | E+G+GE | YES | GC | 2012 | 0.0 | 3.1 | 0.0 | 3.222 | 0.905 | 0.952 |
| Japonica | E+G+GE | YES | GC | 2013 | 0.0 | 1.4 | 0.0 | 0.997 | 2.053 | 1.422 |
| Japonica | E+G+GE | YES | GC | Global | 0.0 | 3.8 | 0.0 | 3.855 | 0.965 | 0.977 |
| Japonica | E+G+GE | YES | PH | 2009 | 18.6 | 0.6 | 19.3 | 0.594 | 0.966 | 0.983 |
| Japonica | E+G+GE | YES | PH | 2010 | 11.9 | 0.6 | 14.2 | 0.671 | 0.838 | 0.915 |
| Japonica | E+G+GE | YES | PH | 2011 | 75.3 | 1.7 | 29.5 | 1.055 | 2.554 | 1.598 |
| Japonica | E+G+GE | YES | PH | 2012 | 137.9 | 2.3 | 151.7 | 2.383 | 0.909 | 0.953 |
| Japonica | E+G+GE | YES | PH | 2013 | 24.3 | 0.8 | 22.3 | 0.786 | 1.091 | 1.044 |
| Japonica | E+G+GE | YES | PH | Global | 53.6 | 1.2 | 47.4 | 1.098 | 1.131 | 1.089 |
| Japonica | E+G | NO | GY | 2009 | 2472032.4 | 2.1 | 3150591.9 | 2.325 | 0.785 | 0.886 |
| Japonica | E+G | NO | GY | 2010 | 5294276.9 | 2.3 | 5635804.7 | 2.418 | 0.939 | 0.969 |
| Japonica | E+G | NO | GY | 2011 | 9254278.9 | 3.6 | 1932673.7 | 1.628 | 4.788 | 2.188 |
| Japonica | E+G | NO | GY | 2012 | 2010191.4 | 1.8 | 2272509.3 | 1.944 | 0.885 | 0.940 |
| Japonica | E+G | NO | GY | 2013 | 380402.0 | 0.9 | 817542.7 | 1.296 | 0.465 | 0.682 |
| Japonica | E+G | NO | GY | Global | 3882236.3 | 2.1 | 2761824.5 | 1.922 | 1.406 | 1.111 |
| Japonica | E+G | NO | PHR | 2009 | 0.0 | 0.9 | 0.0 | 0.874 | 1.000 | 1.031 |
| Japonica | E+G | NO | PHR | 2010 | 0.0 | 1.8 | 0.0 | 1.585 | 1.250 | 1.108 |
| Japonica | E+G | NO | PHR | 2011 | 0.0 | 1.7 | 0.0 | 1.601 | 1.143 | 1.072 |
| Japonica | E+G | NO | PHR | 2012 | 0.0 | 2.5 | 0.0 | 2.519 | 0.964 | 0.984 |
| Japonica | E+G | NO | PHR | 2013 | 0.0 | 0.9 | 0.0 | 0.892 | 1.000 | 0.979 |
| Japonica | E+G | NO | PHR | Global | 0.0 | 1.5 | 0.0 | 1.494 | 1.048 | 1.034 |
| Japonica | E+G | NO | GC | 2009 | 0.0 | 6.8 | 0.0 | 5.550 | 1.500 | 1.228 |
| Japonica | E+G | NO | GC | 2010 | 0.0 | 6.5 | 0.0 | 5.065 | 1.638 | 1.279 |
| Japonica | E+G | NO | GC | 2011 | 0.0 | 1.1 | 0.0 | 0.893 | 1.450 | 1.215 |
| Japonica | E+G | NO | GC | 2012 | 0.0 | 3.2 | 0.0 | 3.218 | 0.972 | 0.986 |
| Japonica | E+G | NO | GC | 2013 | 0.0 | 0.9 | 0.0 | 0.850 | 1.214 | 1.094 |
| Japonica | E+G | NO | GC | Global | 0.0 | 3.7 | 0.0 | 3.115 | 1.178 | 1.187 |
| Japonica | E+G | NO | PH | 2009 | 31.7 | 0.8 | 36.2 | 0.815 | 0.877 | 0.937 |
| Japonica | E+G | NO | PH | 2010 | 17.1 | 0.7 | 14.9 | 0.687 | 1.145 | 1.070 |
| Japonica | E+G | NO | PH | 2011 | 23.8 | 0.9 | 25.4 | 0.979 | 0.939 | 0.969 |
| Japonica | E+G | NO | PH | 2012 | 161.1 | 2.5 | 162.3 | 2.465 | 0.992 | 0.996 |
| Japonica | E+G | NO | PH | 2013 | 33.9 | 1.0 | 45.1 | 1.117 | 0.752 | 0.867 |
| Japonica | E+G | NO | PH | Global | 53.5 | 1.2 | 56.8 | 1.213 | 0.943 | 0.968 |
| Japonica | E+G+GE | NO | GY | 2009 | 3727865.5 | 2.5 | 3204021.6 | 2.345 | 1.163 | 1.079 |
| Japonica | E+G+GE | NO | GY | 2010 | 6840357.8 | 2.7 | 5605103.8 | 2.412 | 1.220 | 1.105 |
| Japonica | E+G+GE | NO | GY | 2011 | 1937358.9 | 1.6 | 2106138.2 | 1.699 | 0.920 | 0.959 |
| Japonica | E+G+GE | NO | GY | 2012 | 3402284.5 | 2.4 | 2240721.1 | 1.930 | 1.518 | 1.232 |
| Japonica | E+G+GE | NO | GY | 2013 | 633483.2 | 1.1 | 926547.8 | 1.380 | 0.684 | 0.827 |
| Japonica | E+G+GE | NO | GY | Global | 3308270.0 | 2.1 | 2816506.5 | 1.953 | 1.175 | 1.059 |
| Japonica | E+G+GE | NO | PHR | 2009 | 0.0 | 0.8 | 0.0 | 0.798 | 1.167 | 1.054 |
| Japonica | E+G+GE | NO | PHR | 2010 | 0.0 | 2.5 | 0.0 | 1.573 | 2.563 | 1.614 |
| Japonica | E+G+GE | NO | PHR | 2011 | 0.0 | 1.2 | 0.0 | 1.604 | 0.524 | 0.720 |
| Japonica | E+G+GE | NO | PHR | 2012 | 0.0 | 2.6 | 0.0 | 2.540 | 1.073 | 1.030 |
| Japonica | E+G+GE | NO | PHR | 2013 | 0.0 | 0.8 | 0.0 | 0.904 | 0.833 | 0.931 |
| Japonica | E+G+GE | NO | PHR | Global | 0.0 | 1.6 | 0.0 | 1.484 | 1.190 | 1.077 |
| Japonica | E+G+GE | NO | GC | 2009 | 0.0 | 3.7 | 0.0 | 5.647 | 0.424 | 0.654 |
| Japonica | E+G+GE | NO | GC | 2010 | 0.0 | 4.5 | 0.0 | 5.300 | 0.733 | 0.853 |
| Japonica | E+G+GE | NO | GC | 2011 | 0.0 | 1.4 | 0.0 | 0.892 | 2.579 | 1.585 |
| Japonica | E+G+GE | NO | GC | 2012 | 0.0 | 2.9 | 0.0 | 3.186 | 0.841 | 0.917 |
| Japonica | E+G+GE | NO | GC | 2013 | 0.0 | 1.1 | 0.0 | 0.863 | 1.786 | 1.321 |
| Japonica | E+G+GE | NO | GC | Global | 0.0 | 2.7 | 0.0 | 3.177 | 0.867 | 0.862 |
| Japonica | E+G+GE | NO | PH | 2009 | 38.5 | 0.8 | 38.2 | 0.837 | 1.007 | 1.004 |
| Japonica | E+G+GE | NO | PH | 2010 | 17.1 | 0.7 | 14.3 | 0.673 | 1.199 | 1.095 |
| Japonica | E+G+GE | NO | PH | 2011 | 208.1 | 2.8 | 25.2 | 0.976 | 8.246 | 2.872 |
| Japonica | E+G+GE | NO | PH | 2012 | 146.9 | 2.3 | 164.7 | 2.484 | 0.892 | 0.944 |
| Japonica | E+G+GE | NO | PH | 2013 | 29.8 | 0.9 | 44.0 | 1.103 | 0.678 | 0.824 |
| Japonica | E+G+GE | NO | PH | Global | 88.1 | 1.5 | 57.3 | 1.215 | 1.538 | 1.257 |
